# Climate-invariant machine learning

**DOI:** 10.1126/sciadv.adj7250

**Published:** 2024-02-07

**Authors:** Tom Beucler, Pierre Gentine, Janni Yuval, Ankitesh Gupta, Liran Peng, Jerry Lin, Sungduk Yu, Stephan Rasp, Fiaz Ahmed, Paul A. O’Gorman, J. David Neelin, Nicholas J. Lutsko, Michael Pritchard

**Affiliations:** ^1^ Faculty of Geosciences and Environment, University of Lausanne, Lausanne, VD 1015, Switzerland.; ^2^ Department of Earth System Science, University of California, Irvine, CA 92697, USA.; ^3^ Department of Earth and Environmental Engineering, Columbia University, New York, NY 10027, USA.; ^4^ Department of Earth, Atmospheric, and Planetary Sciences, Massachusetts Institute of Technology, Cambridge, MA 02139, USA.; ^5^ Google Research, Mountain View, CA 94043, USA.; ^6^ Department of Atmospheric and Oceanic Sciences, University of California, Los Angeles, Los Angeles, CA 90095, USA.; ^7^ Scripps Institution of Oceanography, University of California, San Diego, La Jolla, CA 92037, USA.; ^8^ NVIDIA, Santa Clara, CA 95050, USA.

## Abstract

Projecting climate change is a generalization problem: We extrapolate the recent past using physical models across past, present, and future climates. Current climate models require representations of processes that occur at scales smaller than model grid size, which have been the main source of model projection uncertainty. Recent machine learning (ML) algorithms hold promise to improve such process representations but tend to extrapolate poorly to climate regimes that they were not trained on. To get the best of the physical and statistical worlds, we propose a framework, termed “climate-invariant” ML, incorporating knowledge of climate processes into ML algorithms, and show that it can maintain high offline accuracy across a wide range of climate conditions and configurations in three distinct atmospheric models. Our results suggest that explicitly incorporating physical knowledge into data-driven models of Earth system processes can improve their consistency, data efficiency, and generalizability across climate regimes.

## Teaser

Physically informed transformations aid the machine learning of Earth system model processes that generalize across climates.

## INTRODUCTION

### Background

Following its success in computer vision and natural language processing, machine learning (ML) is rapidly percolating through climate science [e.g., reviews by (*1*–*5*)]. We use the term ML here to broadly describe algorithms that learn a task from data without being explicitly programmed for that task. Applications of ML in atmospheric science include the emulation of radiative transfer algorithms [e.g., (*6*–*9*)], momentum fluxes [e.g., (*10*–*13*)] and microphysical schemes [e.g., (*14*–*16*)], the bias correction of climate predictions [e.g., (*17*, *18*)], the detection and classification of clouds and storms [e.g., (*19*–*22*)], and the development of subgrid-scale “closures” (i.e., representation based on coarse-scale processes only) from high-resolution simulation data [e.g., (*23*–*25*)], which is the main application discussed here.

ML algorithms typically optimize an objective on a training dataset and make implicit assumptions when extrapolating. Here, extrapolation refers to predictions outside of the training data range, henceforth referred to as out-of-distribution predictions. As an example, multiple linear regressions (MLR) assume that the linear relationship that best describes the training set is valid outside of that training set. Alternatively, when confronted with out-of-distribution inputs, random forests (RFs) (*26*) find the closest inputs in their training sets and assign the corresponding outputs regardless of the out-of-distribution input values. Neural networks (NNs), which are powerful nonlinear regression and classification tools, rely on nonlinear activation functions and fitted weights to extrapolate. Except in specific situations (e.g., samples in the close neighborhood of the training set or described by the same nonlinear mapping as the training set), there is no reason why NNs should generalize well far outside of their training sets. We show later that different NN training approaches on the same data can lead to drastically different out-of-distribution predictions, highlighting the uncertainty associated with such predictions.

In climate applications, this extrapolation issue means that ML algorithms typically fail when exposed to dynamic, thermodynamic, or radiative conditions that differ substantially from the range of conditions that they were trained on. Examples include O’Gorman and Dwyer (*27*), who showed that an RF-based moist convection scheme generalizes poorly in the tropics of a climate 6.5 K warmer than the training climate, and Hernanz *et al*. (*28*), who showed that NNs and support vector machines downscaling surface air temperature made substantial extrapolation errors when exposed to temperatures 2-3 K warmer than in the training set. Rasp *et al*. (*29*) showed that an NN-based thermodynamic subgrid-scale closure generalizes well to climates 1 to 2 K warmer than the training one but makes large errors as soon as the test climate is 4 K warmer than the training one. Beucler *et al*. (*30*) confirmed that these generalization errors remain even when the NN subgrid closure is modified to enforce conservation laws to within machine precision. This has led several studies to recommend training ML models in multiple climates if possible (*31*, *32*). Both Guillaumin and Zanna (*33*) who trained an NN parameterization for subgrid oceanic momentum transport and Molina *et al.* (*34*) who trained convolutional NNs (*35*) to classify thunderstorms in high-resolution model outputs found that their ML models generalized well to a warmer climate. While this may be because both models relied heavily on velocity inputs and their gradients, whose distributions changed only slightly when the climate warmed, Molina *et al.* noted that using two types of ML layers, namely, batch normalization (BN) (*36*) followed by dropout (DP) (*37*), was key to this successful generalization.

DP and BN are two examples of a larger set of methods that help NNs generalize and avoid overfitting, broadly referred to as “regularizations” (*38*). Most empirical regularization methods (e.g., L1 regularization) rely on the parsimony principle, i.e., that simpler models, accurately describing the training set with fewer fitted parameters, are preferable to more complex models and generalize better to unseen conditions. More systematic approaches to regularization have been developed to use ML models in out-of-distribution situations that still require the same inputs/outputs, referred to as domain adaptation [e.g., (*39*–*41*)], a particular case of transfer learning [e.g., (*42*)]. While not all domain adaptation approaches (sample-based, statistics-based, ensemble-based, domain-invariant feature learning, domain mapping, etc.) need supervision (*43*), they usually require at least a few samples in the generalization domain.

Without dismissing existing domain adaptation methods, we here focus on physically informed methods that do not require samples in the generalization domain for three reasons: (i) one of the climate science community’s long-term goals is to train ML models that rely on historical observations only as we cannot, by definition, observe the future climate; (ii) as shown later, even if we have access to simulation data across climates, ML models that intrinsically generalize to climates that they have not been trained on tend to be more data-efficient and robust to other changes (e.g., configuration changes); and (iii) physically informed methods can be readily combined with existing domain adaptation and regularization methods. Motivated by these challenges, we ask: How can we enhance ML algorithms with physical knowledge to make accurate predictions in climate conditions that, in standard variables, lie far outside of the training set?

### Problem definition

**Fig. 1.**
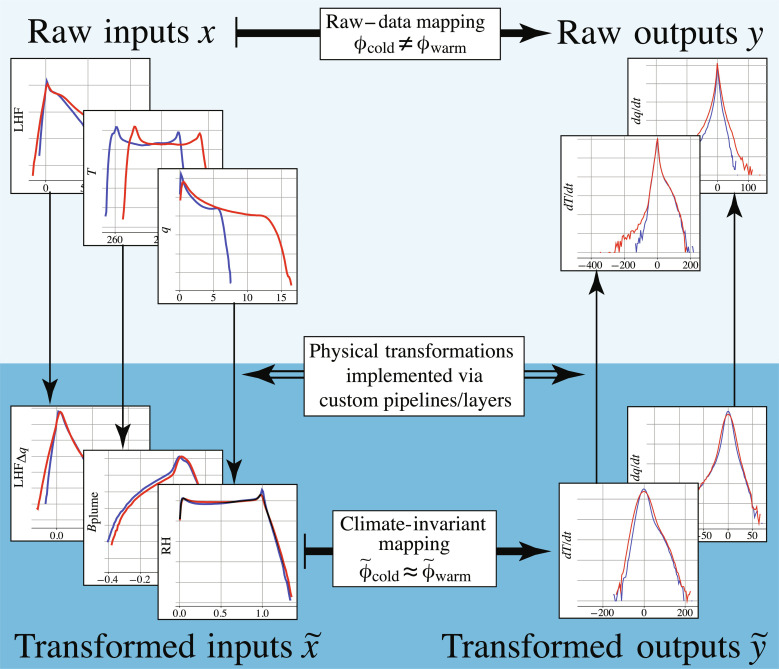
By transforming inputs *x* and outputs *y* to match their probability density functions across climates, the algorithms can learn a transformed mapping $\tilde{\phi }$ that holds across climates. To illustrate this, we show the marginal distributions of inputs and outputs in two different climates using blue and red lines, before (top) and after (bottom) the physical transformation.

Our scientific contribution is to transform a mapping constructed using the original data’s features, henceforth referred to as “raw-data” mapping, into a mapping that remains nearly constant across climates, here referred to as “climate-invariant.” Inspired by invariants in physics and self-similarity in fluid mechanics (*44*), we make the ML-emulated mapping climate-invariant by transforming the input and output vectors so that their distributions shift minimally across different climates (see Fig. 1). We demonstrate this framework’s utility by adapting ML closures of subgrid atmospheric thermodynamics (i.e., coarse-scale thermodynamic tendencies resulting from subgrid convection, radiation, gravity waves, and turbulence) so that they generalize better across climates.

The motivation for this application is twofold. First, purely physically based subgrid closures remain one of the largest sources of uncertainties in Earth system models (*45*–*47*). While ML-based closures have emerged as a promising alternative to traditional semiempirical models (*48*), they lack robustness (*49*, *50*) and, as discussed earlier, usually fail to generalize across climates (*27*, *29*, *30*). Second, atmospheric thermodynamic processes are directly affected by global temperature changes, e.g., in response to anthropogenically forced climate change (*51*). Therefore, predicting subgrid thermodynamics in a warm climate with an ML model trained in a cold climate leads to very apparent failure modes (*30*) that we can transparently tackle.

In mathematical terms, our goal is to build a climate-invariant mapping between the input vector ***x*** representing the large-scale (≈100 km) climate state and the output vector ***y*** grouping large-scale thermodynamic tendencies due to explicitly resolved convection and parameterized radiative transfer and turbulent mixing at the ∼1-km scale [see section SB1 for details]. We keep the overall structure of the mapping ***x*** ↦ ***y*** fixed throughout the manuscript and aim to predict ***y*** as accurately as possible in training and generalization climates (out-of-distribution prediction). Note that this mapping makes some implicit assumptions based on successful past work (*29*, *52*), including locality in horizontal space and time (outputs only depend on inputs in the same atmospheric column at the same time step) and determinism (only one possible output vector for a given input vector). We include cloud radiative effects in all heating terms (total heating $\dot{T}$ , longwave heating **lw**, and shortwave heating **sw**) but, for simplicity, do not predict changes in cloud liquid water and ice and exclude cloud water and greenhouse gases other than water vapor ***q***_***v***_ from the input vector ***x***.

After introducing the climate simulations and training/validation/test split (see Data), we define the climate-invariant mapping and feature transformations (see Theory) and demonstrate and explain their ability to generalize (see Results) before concluding. We refer the reader to the Supplementary Materials for data availability (section SA), additional derivations and descriptions of the mapping and physical transformations (section SB), the implementation of our ML framework (section SC), and additional results (section SD).

## DATA

**Fig. 2.**
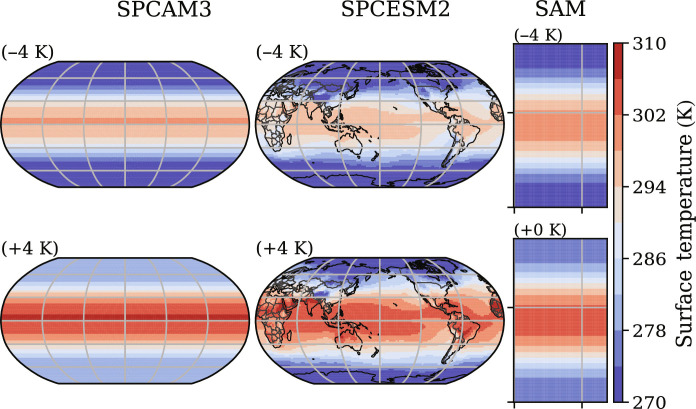
Surface temperatures in the three used atmospheric models. Prescribed surface temperature (in kelvin) for (**left**) the aquaplanet SPCAM3 model and (**right**) the hypohydrostatic SAM model. (**Center**) Annual-mean, near-surface air temperatures in the Earth-like SPCESM2 model.

To test the robustness of our framework across model formulations and configurations, we use three distinct storm-resolving climate models and experimental setups: aquaplanet simulations using the Super-Parameterized Community Atmosphere Model version 3.0 (SPCAM3), Earth-like simulations (i.e., with continents) using the Super-Parameterized Community Earth System Model version 2 (SPCESM2), and quasi-global aquaplanet hypohydrostatic simulations using the System for Atmospheric Modeling version 6.3 (SAM). SPCAM3 and SPCESM2 assume a strict scale separation between the resolved coarse scales and subgrid processes, making them ideal testbeds to machine learn local subgrid closures (*29*, *53*). In contrast, SAM does not assume scale separation as a global storm-resolving model. This improves realism but requires coarse-graining SAM’s output for ML parameterization purposes (*54*, *55*). For each climate model, we run three simulations with three different prescribed surface temperature distributions: (i) (+0 K) a reference simulation with a temperature range analogous to the present climate, (ii) (−4 K) a cold simulation with surface temperatures 4 K cooler than the (+0 K) simulation, and (iii) (+4 K) a warm simulation with surface temperatures 4 K warmer than the (+0 K) simulation, with the exception of SAM for which only the (−4 K) and (+0 K) simulations are available. By prescribing surface temperature, we focus on ML’s ability to consistently predict the atmospheric response to climate change across configurations. Projecting climate change involves a broader range of processes and is beyond this work’s scope. We summarize the simulations and indicate their spatiotemporal resolutions in table S1. Figure 2 gives a visualization of surface temperatures in each model, and fig. S1 provides snapshots of mid-tropospheric subgrid heating, which is one of our ML models’ outputs.

### Super-parameterized aquaplanet simulations

We use data from 2-year SPCAM3 (*56*) climate simulations in an aquaplanet configuration (*57*), with zonally symmetric surface temperatures fixed to a realistic meridionally asymmetric profile (*58*). The insolation is fixed to boreal summer conditions with a full diurnal cycle. A two-dimensional storm-resolving model is embedded in each grid cell of SPCAM3, namely, eight SAM atmospheric columns using a spatiotemporal resolution of 4 km × 30 levels × 25 s and the default one-moment microphysical scheme (*59*). SPCAM3 combines a spectral primitive equation solver with a semi-Lagrangian dynamical core for advection (*57*). The (+0 K) SPCAM3 simulation was first presented in (*53*) and subsequently used to train ML subgrid closures in (*29*, *50*, *60*). Inspired by the generalization experiment of (*27*), the (+4 K) simulation was introduced in (*29*), and we ran the (−4 K) simulation for the work presented here to increase the surface temperature generalization gap from 4 to 8 K.

### Super-parameterized Earth-like simulations

We run three 2-year SPCESM2 (*61*) climate simulations in an Earth-like configuration with realistic surface boundary conditions, including a land surface model, seasonality, aerosol conditions representative of the year 2000, and a zonally asymmetric annual climatology of sea surface temperatures derived from the “HadOIB1” dataset (*62*). We use CESM v2.1.3 to couple CAM v4.0 with the Community Land Model version 4.0 and similarly embed 32 SAM columns in each atmospheric grid cell to explicitly represent deep convection. Our (+0 K) simulation is similar to that in (*52*), which showed the potential of ML for subgrid closures in Earth-like conditions.

### Quasi-global aquaplanet hypohydrostatic simulations

While super-parameterization is well adapted to statistically learning subgrid closures due to its explicit scale separation, this scale separation comes at the cost of distorted mesoscale systems and momentum fluxes (*63*). Furthermore, most ML subgrid closures are based on coarse-graining high-resolution simulations [e.g., (*64*, *65*)]. This motivates us to also test the climate-invariant framework in hypohydrostatic SAM simulations in which the dynamics are not affected by a prescribed scale separation. Computational expense is reduced through hypohydrostatic scaling, which multiplies the vertical acceleration in the equations of motion by a factor of 16 to increase the horizontal scale of convection without overly affecting the larger-scale flow (*66*, *67*). While these simulations use idealized settings, such as aquaplanet configurations, an anelastic dynamical core, a quasi-global equatorial beta plane domain, and perpetual equinox without a diurnal cycle, O’Gorman *et al*. (*68*) showed that they produce tropical rainfall intensity and cluster-area distributions that are close to satellite observations. The prescribed surface temperature distribution in the control simulation of (*68*) is designed to be close to zonal-mean observations (*69*), and its maximum value is roughly 2 K colder than that of the distribution used for the (+0 K) SPCAM3 simulation. To better match the SPCAM3 maxima of distributions of upper-level temperatures and humidities, we choose to treat this SAM control simulation as the (−4 K) SAM simulation and the warm simulation of (*68*) as the (+0 K) SAM simulation. We refer the reader interested in the details of the simulations and the coarse-graining (here by a factor of 8) to (*54*). Differences in climate model formulation and ML parameterization design lead to key differences in the mappings learned for SAM as compared to SPCAM3/SPCESM2, which we summarize below: (i) The input vector does not contain specific humidity, surface pressure, sensible heat fluxes, or latent heat fluxes (LHFs) but instead contains the total non-precipitating water concentration and uses distance to the equator as a proxy for solar insolation; (ii) the output vector includes the subgrid total non-precipitating water tendency instead of the subgrid specific humidity tendency and the subgrid liquid/ice static energy tendency instead of the subgrid temperature tendency; (iii) the output vector does not contain subgrid longwave and shortwave heating; (iv) SAM uses a height-based vertical coordinate rather than a pressure-based one; and (v) the generalization experiment is from (−4 K) to (+0 K) [unavailable (+4 K) simulation].

### Normalization and training, validation, and test split

Both generalization experiments expose ML models to out-of-distribution inputs that they have not been trained on. Following best ML practices (*70*), we use the training set to optimize the ML model’s trainable parameters, save the trainable parameters that led to the best performance on the validation set to avoid overfitting the training set, and evaluate the final model on samples from a separate test set. We split each of the eight simulations into training/validation/test sets by using noncontiguous 3-month periods (reported in table S1) to avoid high temporal correlations between training/validation/test set samples (*71*). Following (*29*), the normalization procedure involves subtracting the mean value of each input variable at each vertical level and dividing by the maximum range of that variable across the entire atmospheric column.

To understand which solutions are most promising for helping ML algorithms generalize to unseen conditions, we design two generalization experiments: (i) training and validating ML models on cold simulations (−4 K) and testing them on warm simulations (+4 K for SPCAM3/SPCESM2 and +0 K for SAM); and (ii) training and validating ML models on aquaplanet simulations (SPCAM3) and testing them on Earth-like simulations with continents (SPCESM2).

## THEORY

We formally define a climate-invariant mapping as a mapping that is unchanged across climates. In practice, it is difficult to find mappings that are exactly invariant, and we will use the terminology climate-invariant for any mapping that remains approximately constant across climates. To achieve climate invariance, we introduce physically based feature transformations, defined as physically informed functions that map the inputs/outputs to different inputs/outputs whose distributions vary little across climates. We deem the physical transformation to be climate-invariant if it is successful at limiting distributions variations of the inputs/outputs across climates. Note that climate-invariant transformations are distinct from nondimensionalization in dimensional analysis, as nondimensionalization does not necessarily alter distribution shape while climate-invariant transformations may yield variables that have physical units.

**Fig. 3.**
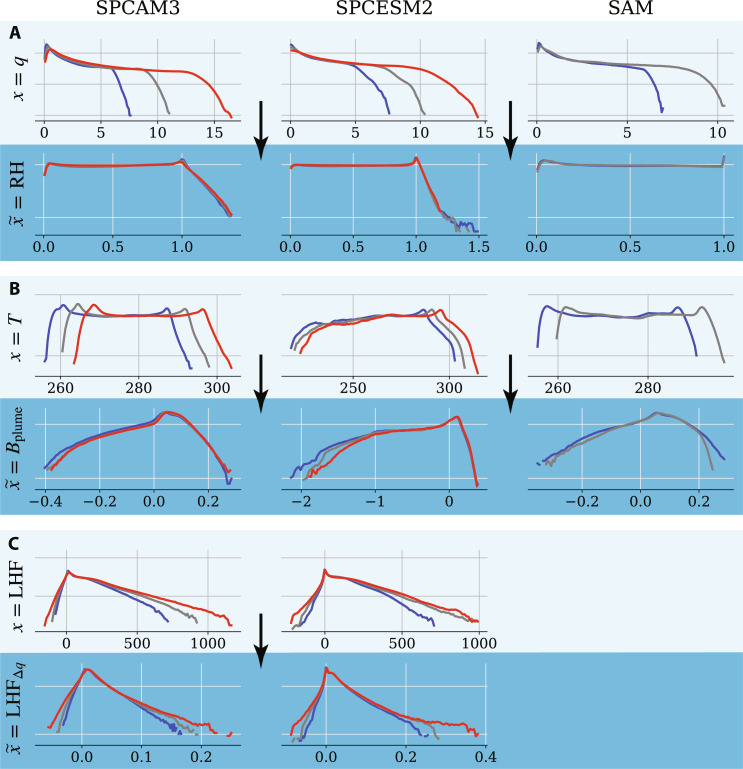
Physical transformations can align distributions across climates. We show the univariate distributions of selected raw inputs ***x***: (**A**) 600-hPa specific humidity; (**B**) 850-hPa temperature; and (**C**) latent heat flux (LHF) in the cold (blue), reference (gray), and warm (red) simulations of each model (SPCAM3, SPCESM2, and SAM). For each variable, we also show the PDFs of the transformed inputs $\tilde{x}$ as discussed in the Theory section. From top to bottom, the variables are *q* (grams per kilogram), relative humidity (RH), *T* (kelvin), *B*_plume_ (meters per square second), LHF (watts per square meter), and LHF_Δ*q*_ (kilograms per square meter per second). For a given variable and transformation, we use the same vertical logarithmic scale across models.

Throughout the following section, we compare two transformation options for each input, whose univariate Probability Density Functions (PDFs) are depicted for all three atmospheric models in Fig. 3: no transformation (top) and our most successful transformation (bottom). All transformations are derived in section SB2. Our comparison relies on the Hellinger and Jensen-Shannon PDF distance metrics defined and calculated in Materials and Methods and section SD1. To prevent information leaks from generalization test sets into the physically informed ML framework, we take two precautions: (i) the physical transformations are fixed, meaning that their structure and parameters are non-trainable; and (ii) transformations are ranked on the basis of their generalization from (−4 K) to (+0 K) in SPCAM3. Our (+4 K) results across models and configurations independently confirm this ranking.

### Specific humidity

Without any transformation, the PDF of specific humidity ***q*** (Fig. 3A, top) extends through a considerably larger range as the climate warms. This is because, barring supersaturation, ***q*** has a theoretical upper bound in a given climate, namely, the saturation specific humidity, which increases quasi-exponentially with temperature through the Clausius-Clapeyron relation [e.g., (*72*, *73*)]. The relative humidity (RH) transformation ${\tilde{q}}_{\text{RH}}$ (Fig. 3A, bottom) normalizes specific humidity by its saturation value. As a result, most of the RH PDF lies within [0,1], except for a few atmospheric columns exhibiting supersaturation in SPCAM, and that PDF changes little as the climate warms (*74*). In addition to capturing grid-scale saturation, ${\tilde{q}}_{\text{RH}}$ helps predict the subgrid effects of dry-air entrainment, known to regulate tropical convection (*75*, *76*, *77*) (see section SB2b for details of RH calculations).

### Temperature

The PDF of temperature ***T ***(Fig. 3B, top) shifts quasi-linearly as the climate warms. To address this shift without compromising the approximate invariance of tropopause temperatures with warming (*78*, *79*, *80*), we derive a temperature transformation directly relevant for moist convection: the buoyancy of a non-entraining, moist static energy-conserving plume ${\tilde{T}}_{\text{buoyancy}}$ (Fig. 3B, bottom, see section SB2c for this buoyancy’s derivation). This transformation is inspired by recently introduced lower-tropospheric buoyancy measures (*81*, *82*), but with an extension to the full troposphere (*83*). While ${\tilde{T}}_{\text{buoyancy}}$ does not explicitly include entrainment effects, the mapping of ${\tilde{T}}_{\text{buoyancy}}\left(p\right)$ and ${\tilde{q}}_{\text{RH}}\left(p\right)$ to heating and moisture sink will implicitly include these. This transformation captures leading order effects needed to yield approximate climate invariance (Fig. 3B). ${\tilde{T}}_{\text{buoyancy}}$ increases physical interpretability by linking the vertical temperature structure and near-surface humidity changes to a metric that correlates well with deep convective activity (*84*). ${\tilde{T}}_{\text{buoyancy}}$ also captures the role of near-surface humidity relative to the temperature structure aloft in contributing to moist convective instability in the tropics.

### Latent heat flux

The last input whose distribution changes visibly with warming is the LHF (Fig. 3C, top; the remaining inputs, sensible heat flux and surface pressure, change less with warming and are discussed in section SB2d). Similar to specific humidity, the increase of LHFs with warming is directly linked to the Clausius-Clapeyron relationship [e.g., (*85*)]. To address this shift, we leverage the bulk aerodynamic formula to represent surface fluxes and to provide a physics-motivated transformation of LHF using the near-surface saturation deficit (Fig. 3C, bottom). This transforms LHF, a thermodynamic variable, into $L\tilde{H}F_{\Delta q}$ , approximately proportional to the magnitude of near-surface horizontal winds and density [e.g., (*85*)], whose distributions vary less with warming. Note that this scaling is less effective over land (e.g., in SPCESM2) where evapotranspiration changes do not follow a Clausius-Clapeyron scaling.

We now show that all three input transformations $[{\tilde{q}}_{\text{RH}}\left(p\right)$ , ${\tilde{T}}_{\text{buoyancy}}\left(p\right)$ , and $L\tilde{H}F_{\Delta q}$ ] lead to statistically significant improvements in the ML models’ ability to generalize.

## RESULTS

**Fig. 4.**
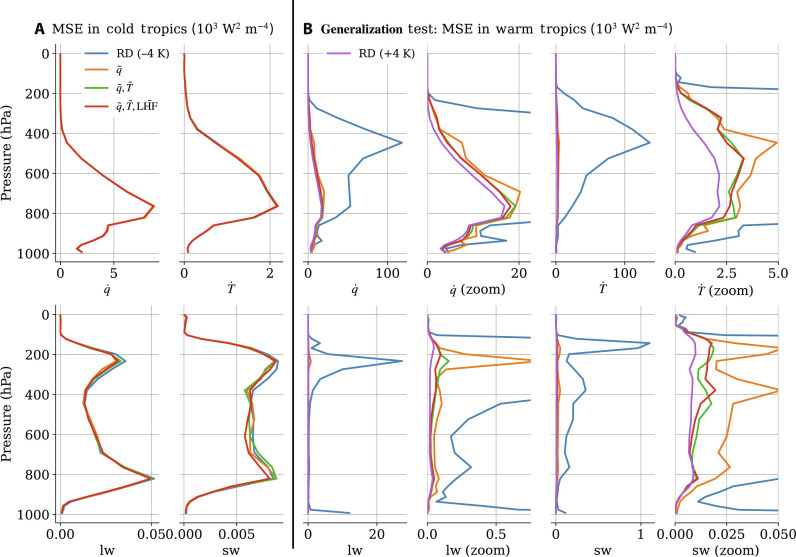
All neural networks (NNs), trained in the cold climate, exhibit low error in the cold climate’s test set, but much larger error in the warm climate’s test set. (A) Low error in the cold climate’s test set. (B) Larger error in the warm climate’s test set. This generalization error decreases as inputs are incrementally transformed: first no transformation (blue), then the vertical profile of specific humidity (orange), then the vertical profile of temperature (green), and lastly LHFs (red). For reference, the purple line depicts an NN trained in the warm climate. We depict the tendencies’ mean-squared error (MSE) versus pressure, horizontally averaged over the tropics of SPCAM3 aquaplanet simulations, for the four model outputs: total moistening ( $\dot{q}$ ), total heating ( $\dot{T}$ ), longwave heating (lw), and shortwave heating (sw). Given that the raw-data NN’s generalization error (blue line) greatly exceeds that of the transformed NNs, we zoom in on each panel to facilitate visualization.

**Fig. 5.**
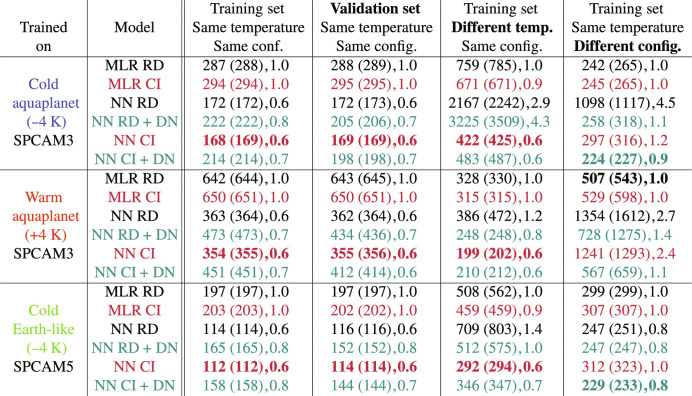
Model error across temperatures and configurations. MSE (in W^2^ m^−4^) of six models trained in three simulations (first column) and evaluated over the training or validation set of the same and two other simulations (last four columns). The models (second column) are raw-data (RD) or climate-invariant (CI), and MLRs or neural nets (NN), and sometimes include DP layers preceded by a BN layer (DN). The models are trained for 20 epochs. We first provide the MSE corresponding to the epoch of minimal validation loss, then the MSE averaged over the five epochs with lowest validation losses (in parentheses), and lastly the MSE divided by the baseline MSE, where we use the raw-data MLR as baseline. Note that “different temperature” refers to (+4 K) for (−4 K) training sets and vice versa. In each application case, we highlight the best model’s error using bold font.

**Fig. 6.**
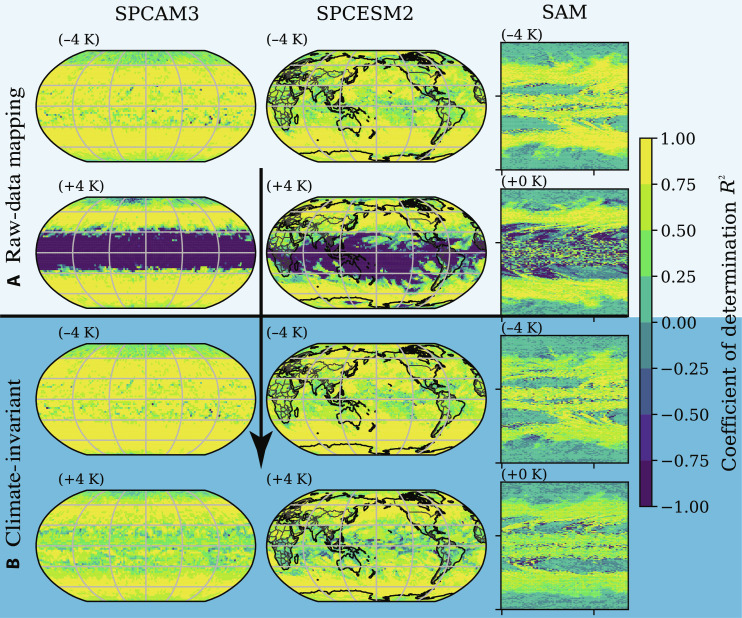
Climate-invariant NNs address the raw-data NNs’ generalization problems in the warm tropics. This is demonstrated by the 500-hPa subgrid heating’s coefficient of determination R_2 _calculated over the test set for the raw-data (A) and climate-invariant (B) NNs. We train NNs using the cold (−4 K) training set of each model (SPCAM3, SPCESM2, and SAM). We note that these NNs do not use DP nor BN, and we refer the readers to fig. S8 for latitude-pressure cross sections.

**Fig. 7.**
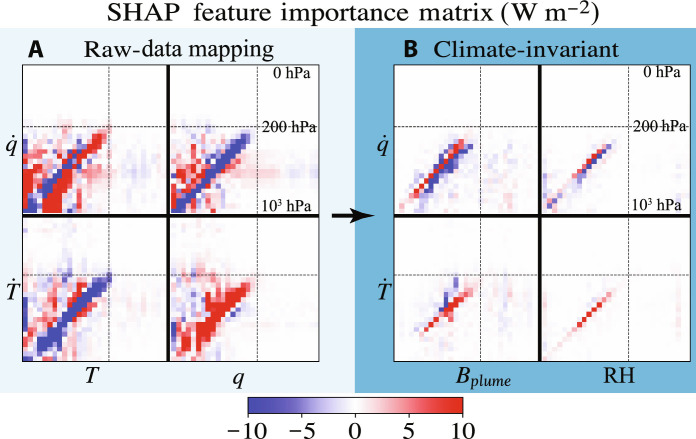
Explainable artificial intelligence suggests that climate-invariant mappings are more spatially local. We depict *ℳ* for the (**A**) raw-data and (**B**) climate-invariant NNs trained in the SPCAM3 (+4 K) warm aquaplanet simulation. The *x* axes indicate the inputs’ vertical levels, from the surface (left, 10^3^ hPa) to the top of the atmosphere (right, 0 hPa), while the *y* axes indicate the outputs’ vertical levels, from the surface (bottom, 10^3^ hPa) to the top of the atmosphere (top, 0 hPa). We additionally indicate the 200-hPa vertical level with black dashed lines.

**Fig. 8.**
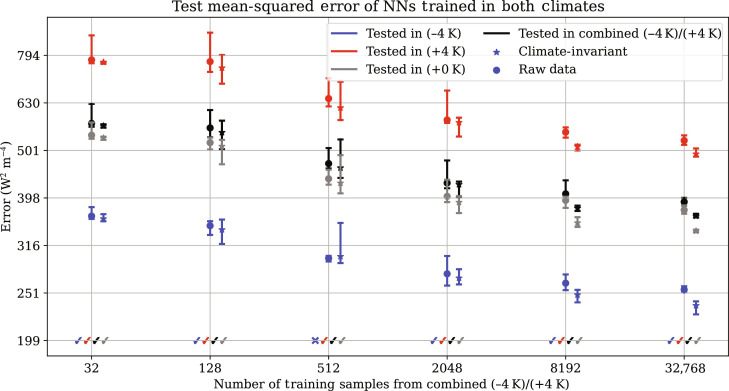
Climate-invariant (CI) NNs trained on datasets containing both cold (−4 K) and warm (+4 K) samples outperform raw-data (RD) models offline in *≈*95% of cases, with less sensitivity to the data partition used for training. Dots on the left represent the median RD error from a 10-fold cross-validation without replacement, with horizontal ticks indicating the first and ninth deciles. Stars on the right correspond to the median CI error. Ticks denote the majority of cases, for which CI models outperform RD models, even when data from both climates are available; crosses indicate the rare exceptions. We use a logarithmic scale for both axes.

The results are organized as follows. After demonstrating the benefits of progressively transforming the ML models’ inputs (Fig. 4), we show how climate-invariant models learn subgrid closures across climates and configurations during training (Fig. 5 and fig. S7). We then discuss the global skill of different models after training (Fig. 6 and figs. S8 and S9). Last, we investigate the structure of climate-invariant mappings to understand why they generalize better across climates (Fig. 7), even when data from multiple climates are available (Fig. 8).

### Benefits of incremental input transformations

In this section, we demonstrate that incrementally transforming the inputs of NNs progressively improves their generalization abilities from the cold (−4 K) aquaplanet (SPCAM3) simulation to the warm (+4 K) aquaplanet simulation. The largest surface temperature jump tested in this study is between the cold aquaplanet simulation and the tropics of the warm aquaplanet simulation (“warm tropics” for short), defined as the regions with out-of-distribution surface temperatures, whose latitudes are between −15°S and 23°N (approximately the red regions in top-left subplots in Fig. 2). To expose the failure modes of the “brute force” model and the benefits of progressively transforming the inputs, we first trained several NNs on the cold aquaplanet until they reached high accuracy (Fig. 4A) before testing their out-of-distribution generalizability in the warm tropics (Fig. 4B).

In the cold tropics, the vertical profiles of the mean-squared error (MSE) are nearly indistinguishable for all types of NNs and roughly follow the vertical structure of subgrid variance, as discussed in (*53*, *60*). When evaluated in the warm tropics, the MSE of the brute force NN (blue line) increases by a factor of ≈10 and peaks above 100 W^2^ m^−4^, underlining how raw-data NNs fail to generalize across climates. As discussed in Theory, we progressively transformed the inputs starting with specific humidity, which is transformed to RH (orange line). This first transformation decreases the MSE so much (by a factor of 5 to 10) that we need to zoom in on each panel to distinguish the generalization abilities of additional NNs. Adding the transformations of temperature to plume buoyancy (green line) improves the generalization MSE for all variables. Adding the LHF to $L\tilde{H}F_{\Delta q}$ transformation (red line) further decreases generalization MSE, except for shortwave heating where the MSE improves in the cold but not the warm climate. Impressively, transforming all three inputs decreases the MSE so much that the resulting climate-invariant NN’s MSE (red line) is within ≈25% of the MSE of a raw-data NN that was directly trained in the warm climate (purple line).

Hereafter, we use climate-invariant to refer to models for which all three inputs (*q*, *T*, and LHF), but no outputs were transformed, solely based on physical principles. After demonstrating their success in the aquaplanet case, we are now ready to investigate how these climate-invariant models learn in the other climates and simulations introduced in Data.

### Learning across climates and configurations

In this section, we show that climate-invariant models learn mappings that are valid across climates and configurations and that their efficacy improves when used in conjunction with ML regularization techniques like BN and DP layers.

Figure 5 shows the MSE of ML models trained in three different datasets and evaluated over their training and validation sets and over test sets of different temperature and configuration. As discussed previously, climate-invariant NNs (NN CI) generalize better to warmer climates than raw-data NNs (NN RD). We go one step further by examining learning curves, defined as the MSE of an ML model at the end of each epoch during training (one epoch corresponds to the ML model being fed the entire training set once). Impressively, the learning curve of the climate-invariant NN trained in the cold aquaplanet but tested in the warm aquaplanet (starred blue line in fig. S7A) is mostly decreasing, supporting this manuscript’s key result: Climate-invariant NNs continuously learn about subgrid thermodynamics in the warm aquaplanet as they are trained in the cold aquaplanet. In contrast, the raw-data NN trained in the cold aquaplanet but tested in the warm aquaplanet makes extremely large generalization errors, which worsen as the model is trained in the cold aquaplanet (see section SD2 for details).

Climate-invariant NNs also facilitate learning across configurations, i.e., from the aquaplanet to the Earth-like simulations and vice versa (see NN CI rows in Fig. 5). Climate-invariant transformations additionally improve the MLR baseline’s generalization ability, albeit less markedly. This smaller improvement in MLR’s generalization abilities is linked to its relatively small number of trainable parameters, resulting in (i) raw-data MLRs generalizing better than raw-data NNs; and (ii) MLRs having lower descriptiveness and fitting their training sets less well, limiting the maximal accuracy of climate-invariant MLRs on test sets.

There are a few cases in which transforming inputs does not fully solve the generalization problem, e.g., when trying to generalize from the aquaplanet to the Earth-like simulation. In that case, we leverage the fact that input transformations can easily be combined with standard techniques to improve generalization, such as DP layers before each activation function and a single BN layer before the first DP layer (*36*). As DP layers randomly drop a fixed proportion of the trainable parameters during training, NNs with DP fit their training set less well (see NN CI + DN row of Fig. 5). However, they improve generalization in difficult cases (e.g., between cold aquaplanet and Earth-like simulations) and do not overly deteriorate generalization in cases where the input transformations work particularly well (e.g., from cold to warm aquaplanet). Our results suggest that combining physics-guided generalization methods (e.g., physical transformation of the inputs/outputs) with regularization methods (e.g., DP) is advantageous and deserves further investigation. After analyzing the overall MSE during training, we now turn to the spatial characteristics of our ML models’ skill after training.

### Global performance after training

In this section, we first compare the spatial characteristics of the brute force and climate-invariant NNs’ skill across climates of different temperatures to further establish the advantages of our climate-invariant transformation. These advantages are clearly visible in Fig. 6, where the raw-data models struggle to generalize to the warm tropics for all simulations despite fitting the cold training set well (Fig. 6A). We can trace these generalization errors to warm temperature and moist atmospheric conditions the NNs were not exposed to during training, visible when comparing Fig. 6A with Fig. 2A. In contrast, the climate-invariant models fit the warm climate almost as well as the cold climate that they were trained in (Fig. 6B). Note that Fig. 6 focuses on the horizontal map of a single output, i.e., the total subgrid heating at 500 hPa, but that the horizontal maps of other outputs, such as the near-surface subgrid heating (see fig. S9), all exhibit the same pattern of raw-data models failing in the warm tropics and the climate-invariant models mostly correcting these generalization errors. Last, the spatial distribution of the skill in the training set (e.g., middle column of fig. S8) is reassuringly consistent with the skill map of highly tuned NNs trained in similar conditions [e.g., (*52*)]. This confirms that the raw-data models, representative of state-of-the-art ML subgrid closures, fail to generalize. This failure is confirmed in the latitude-pressure map of the subgrid heating at all vertical levels shown in fig. S8 and discussed in section SD3.

To fully compare ML models across climate and configurations, we evaluate their overall MSEs in the training, validation, and both generalization test sets in Fig. 5. In addition to the MSE of minimal validation loss, we show the MSE averaged over the five epochs of minimal validation loss in parentheses to confirm that our models have converged. Consistent with the learning curves in fig. S7, climate-invariant NNs with DP and BN layers often demonstrate the highest level of generalizability (two rightmost columns of each row’s NN CI + DN models).

While they fit their training sets less well, raw-data MLRs generalize better than raw-data NNs because they have fewer trainable parameters (see MLR RD and NN RD models). In Fig. 5, we also show that, while DP and BN layers generally increase the generalization performance of raw-data NNs (NN RD + DN models), we can systematically improve these standard ML regularization methods by combining them with input transformations (NN CI + DN models).

### Understanding climate invariance

To interpret our NN results, we use a game theory–based explainable ML approach, called SHapley Additive exPlanations (SHAP) (*86*, *87*), to dissect climate-invariant mappings and provide insight into why they generalize better across climates and configurations. Note that MLRs are interpretable by construction, and we can draw preliminary conclusions by visualizing MLR weights without the need for explainable ML libraries (see section SD4). Similarly, we can directly visualize the NNs’ linear responses (*80*, *88*–*90*) by calculating their Jacobians (gradients) via automatic differentiation (*49*). However, the difference between RD and CI MLRs is small and the Jacobians (*88*) cannot always be reliably used to explain nonlinear NN predictions as they are first-order derivatives calculated over a sample (*91*).

Therefore, as climate-invariant NNs have shown superior generalizability from cold to warm climates (see NN CI errors in Fig. 5), we use SHAP’s KernelExplainer to elucidate the NNs’ generalizability. We choose this attribution method for its versatility, as it can be used for any multi-input/output ML model. SHAP estimates the impact of a particular input value *x_i_* on each output *y_j_* of our model. It is designed with a local accuracy property, ensuring that the sum of the effects of individual inputs equals the difference between *y_j_* and its average value in the training set

$$\sum_{i}\text{SHAP}\left(x_{i},y_{j}\right)\overset{\text{def}}{=}{y\prime }_{j}$$ (1)

where we have introduced the deviation ${y\prime }_{j}$ , defined as the difference between *y_j_* and its training set average $y_{i}^{\prime }\overset{\text{def}}{=}y_{i}-{\left\langle y_{i}\right\rangle }_{ℰ}$ . We use these “Shapley values” to build a nonlinear feature matrix *M* capturing the influence of an input *x_i_* on an output *y_j_*

$${ℳ}_{\text{ij}}\overset{\text{def}}{=}{\langle \text{sign}\left({x\prime }_{i}\right)\times \text{SHAP}\left(x_{i},y_{j}\right)\rangle }_{ℰ}$$ (2)

where we use the sign of the input deviation $x_{i}^{\prime }$ to make ${ℳ}_{ij}$ positive if ${x\prime }_{i}$ and ${y\prime }_{j}$ have the same sign, e.g., if a positive input deviation leads to a positive output deviation. In the particular case of the MLR defined in eq. S25, the nonlinear feature matrix becomes the regression weight matrix multiplied by the absolute value of the input deviations: ${ℳ}_{\mathrm{ij}}={\langle A_{\mathrm{ij}}\;|\;{x\prime }_{i}\;|\;\rangle }_{ℰ}$ , confirming that the feature matrix *ℳ* is a nonlinear extension of the Jacobian (*A* in the MLR case).

In Fig. 7, each panel depicts the SHAP feature matrix *M* for a given model: raw-data (A) and climate-invariant (B). Each model’s inputs (e.g., specific humidity *q* and temperature *T*) are organized on the *x* axis from the surface to the top of the atmosphere. Each model’s outputs [subgrid moistening $\dot{q}$ and subgrid heating $\dot{T}$ ; see fig. S14 and fig. S15 for subgrid longwave heating (lw) and subgrid shortwave heating (sw)] are organized on the *y* axis, from the surface to the top of the atmosphere. Following a horizontal line shows how different inputs contribute to a given output, while following a vertical line shows how a given input influences different outputs.

Figure 7 contains a wealth of information about subgrid closures trained in aquaplanet simulations; we focus here on visualizing how the climate-invariant NNs (B) operate in ways that generalize better than their raw-data mapping counterpart (A). Consider the row for subgrid heating  $\dot{T}$ . In the raw-data case (A), *M* has large coefficients in most of the troposphere (in the entire square below the dashed lines depicting the approximate tropopause level). This means that specific humidity and temperature deviations at all levels affect subgrid heating at a given level, i.e., there are large nonlocal relations in the vertical. Some nonlocal relations are physically plausible for convection because buoyant plumes tend to rise from the surface, and near-surface *T* and *q* influence $\dot{T}$ through the entire troposphere. However, in this model, moisture at higher altitudes appears to influence $\dot{q}$ at lower altitudes, raising suspicions that some of the raw-data NN’s nonlocalities are not causal but rather due to high autocorrelations within the input’s vertical profile, as Brenowitz *et al*. (*49*) showed could happen. Temperature variations are observed to have strong vertical correlations (*92*) in part because of deep convective effects. Because temperature affects the saturation threshold for moisture, the RD NN will have to correctly capture the effects of both temperature and moisture wherever either has influence. In contrast, in the *B*_plume_-RH climate-invariant case (B), leading nonlocal effects between the boundary layer and the free troposphere have already been taken into account in the buoyancy formulation, and the temperature-dependent saturation threshold is built into RH. Thus, *M* for $\dot{T}$ tends to be concentrated near the red diagonal, meaning that positive deviations of plume buoyancy and RH increase subgrid heating near the same vertical level. The use of domain knowledge has effectively reduced the effects that must be estimated by the NN for the climate-invariant models. This tends to yield differences between the models trained in the cold and the warm climates that are much smaller than for the raw-data models (see last column of fig. S15).

### Advantages of climate invariance with multi-climate data

It is natural to wonder whether the benefits of climate invariance carry over to training scenarios that entail data from multiple simulations spanning diverse temperatures. In contrast, until now, we have only trained ML models on single-climate simulations. To find out, we examine the benefits of our climate-invariant transformation approach under the ideal scenario where we have access to data from multiple climates. We conduct experiments where we train NNs on both the cold (−4 K) and warm (+4 K) aquaplanet simulations. We progressively increase the amount of training data to assess data efficiency. Throughout the experiment, we use eight batches and systematically increase the batch size by powers of 2, starting with a batch size of 4. Note that we obtain similar results when increasing the number of batches while keeping the batch size fixed (not shown). To obtain well-defined uncertainty estimates, we use a 10-fold cross-validation procedure via random sampling without replacement. Our findings, depicted in Fig. 8, demonstrate that CI NNs (i) consistently outperform RD NNs, particularly in data-rich scenarios; and (ii) exhibit lower sensitivity to the training data partition, resulting in more reliable offline performance with reduced variability in test errors. This confirms that our climate-invariant mapping enhances data efficiency, performance, and fit reproducibility across different climates, even when training data from multiple climates are available.

## DISCUSSION

In the context of climate change, we hypothesized that ML models emulating climate-invariant mappings (Fig. 1), for which the inputs/outputs distributions change little across climates (Fig. 3), generalize much better than ML models emulating raw-data mappings, for which the inputs/outputs distributions change substantially across climates. Tested on a suite of storm-resolving atmospheric simulations with different surface temperatures in three atmospheric models with distinct configurations (Fig. 2), physically transformed NNs generalize better as their inputs are progressively transformed (Fig. 4). Climate-invariant NNs whose inputs have all been transformed learn mappings that are robust to temperature and configuration changes (Fig. 5) and hence exhibit superior generalization skill almost everywhere on the globe (Fig. 6), including when data from multiple climates are available (Fig. 8). Last, attribution maps reveal that in addition to providing control on the features’ distributions, climate-invariant NNs learn more spatially local mappings that facilitate generalization across climates and configurations (Fig. 7).

From a computational perspective, incorporating physical knowledge, here of climate change, into an ML framework to improve its generalization skill is a successful example of using domain knowledge to extract more informative predictors, informally referred to as “feature engineering” [e.g., (*93*)]. This also aids interpretability of the mapping. From a climate science perspective, requiring that a nonlinear statistical model of the atmosphere generalize across climate is a stringent test that helped us discover new mappings. This climate-invariant mapping is more robust to climate and configuration changes and is more advantageous than directly using model and observational outputs (e.g., specific humidity and temperature), even when data are available in various climate regimes. In the particular case of subgrid thermodynamics, our generalization results suggest the possibility of NN-powered closures that could work in Earth-like settings, even in vastly different climate conditions. Last, the attribution maps suggest the possibility of new analytic representations of convection from data, facilitated by the more local climate-invariant representation of subgrid thermodynamics. Our strategy paves the way for the successful use of ML models for climate change studies.

## MATERIALS AND METHODS

**Fig. 9.**
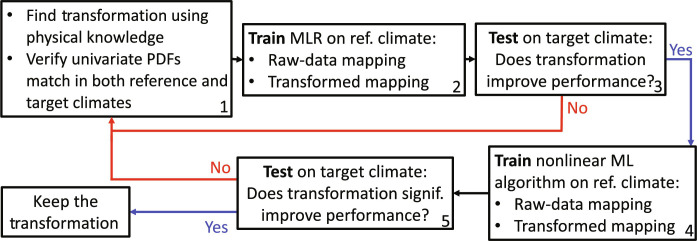
Proposed five-step workflow to find climate-invariant transformations. The transformations help ML models generalize from a reference (ref.) climate to a target one, using (top) a baseline MLR as an initial guide.

This section outlines how to find feature transformations yielding climate invariance. Figure 9 illustrates our proposed workflow for finding robust input/output transformations that transform the initial raw-data mapping into a climate-invariant mapping when combined. Note that this workflow assumes that we cannot or do not want to retrain ML algorithms in the target climate, which excludes automatically finding a transformation by training a model. This limitation could arise because the data in the target climate are insufficient or less reliable or because we seek to uncover new physical relations that hold across an even wider range of climates.

The first step is to propose a physical transformation to implement. We can do this through knowledge of robust physical or statistical relations that link and/or preserve distributions (e.g., state equations, self-similarities, conservation laws, and accurate empirical relations) as modeled in section SB2. These relations help derive invariants [e.g., (*94*)] under a change in thermodynamic conditions. Before taking the time to implement this transformation in the ML workflow, we can verify that the PDFs of the transformed inputs/outputs (approximately) match in the training and target climates. Ideally, the joint PDFs of the transformed inputs/outputs would match. In practice, because it is easier to transform one variable at a time and the data are often insufficient in the target climate, we can fall back on the necessary (but not sufficient) condition that the univariate PDFs of the transformed inputs/outputs must match in the training and target climates. Mathematically, this match can be quantified using PDF distance metrics.

An additional challenge is that the original and transformed variables may have different units and range, meaning that any nonlinear distance metric will complicate the PDF comparison. To address this, we normalize the PDFs and their support variables *X* so that the PDFs’ domains strictly lie within [0,1]. For a given variable, we use the same normalization factors across climates

$$X_{\text{norm}}\overset{\text{def}}{=}\frac{X-{\text{min}}_{\text{cl}}X}{{\text{max}}_{\text{cl}}X-{\text{min}}_{\text{cl}}X}$$ (3)

$$\text{PD}F_{\text{norm}}\overset{\text{def}}{=}\frac{\text{PDF}}{\int_{0}^{1}dX_{\text{norm}}\times \text{PDF}\left(X_{\text{norm}}\right)}$$ (4)

where PDF_norm_ is the transformed PDF and *X*_norm_ is its transformed support; and max_cl_ and min_cl_, respectively, refer to the maximum and minimum operators over the variables’ domains and across climates, i.e., over the (−4 K), (+0 K), and (+4 K) simulations.

Once the PDFs of each variable are normalized, we may pick any informative PDF distance metric to quantify how PDFs match across climates. Here, we pick the commonly used Hellinger distance between two PDFs *p* and *q*, formally defined (*95*) as

$$ℋ\left(p,q\right)\overset{\text{def}}{=}\sqrt{\int_{0}^{1}\frac{\mathrm{dx}}{2}{\left[\sqrt{p\left(x\right)}-\sqrt{q\left(x\right)}\right]}^{2}}$$ (5)

This distance is symmetric (i.e., the arguments’ order does not affect the outcome) and easy to interpret: *ℋ*(*p*, *q*) is bounded by 0 (when *p* = *q*) and 100% (when *p* is zero whenever *q* is positive and vice versa). In section SD1, we show that our results using the Hellinger distance (see table S1) are consistent with those using the Jensen-Shannon distance (see table S3) (*96*), a PDF distance metric giving large weights to the PDFs’ tails that tend to be particularly problematic for generalization purposes.

Once the univariate PDFs of the physically transformed variables match across climates, the second step is to train two inexpensive or “baseline” models on the reference climate to quickly check whether the transformation improves an ML model’s generalization ability: (i) a raw-data model without the transformation and (ii) a climate-invariant model with the transformation. If the transformation does not improve the baseline model’s generalization abilities [i.e., (ii) performs worse than (i) in the target climate], then the transformation may not be appropriate. Note that we trained MLR baselines to create climate-invariant NNs, but the ML model used to define the baseline should be tailored to the desired final ML model.

If the transformation improves the inexpensive baseline model’s performances, then the last step is to train the raw-data and climate-invariant versions of the desired ML model (usually nonlinear) on the reference climate. If the physical transformation improves the desired ML model’s generalization abilities (i.e., the climate-invariant model beats the raw-data model in the target climate using the same performance metric calculated over a validation set), then we may keep the transformation. This workflow may be repeated for the ML model’s additional input/output variables until the emulated mapping is as climate-invariant as possible.

Before applying this workflow to subgrid thermodynamics closures, we underline one of its key challenges: Because some transformations are much more impactful than others, it is often not possible to develop each physical transformation independently. In our case, the specific humidity inputs vary the most across climates, meaning that transforming specific humidity affects the model’s generalization abilities the most. As a result, initial experiments that independently tested the effect of transforming temperature suggested a negative impact of temperature transformation on generalization ability (not shown). This initial result was later invalidated by experiments that jointly transformed specific humidity and temperature. Following this, we adopt a progressive input transformation approach, where the most important inputs are transformed first: specific humidity, then temperature, and lastly surface energy fluxes.

## Supplementary Materials

### This PDF file includes:

Supplementary Text Figs. S1 to S15 Tables S1 to S3 References
