# Supplementary material for: Climate-invariant machine learning
Source: Sci Adv. 2024 Feb 7;10(6):eadj7250. doi: 10.1126/sciadv.adj7250 (PMC10849594; doi:10.1126/sciadv.adj7250)
Supplement: Supplementary file 1 — Supplementary Text Figs. S1 to S15 Tables S1 to S3 References [file sciadv.adj7250_sm.pdf]

Science Advances
AAAS

# Supplementary Materials for

## Climate-invariant machine learning

Tom Beucler *et al.*

Corresponding author: Tom Beucler, tom.beucler@gmail.com

*Sci. Adv.* **10**, eadj7250 (2024)
DOI: 10.1126/sciadv.adj7250

**This PDF file includes:**

Supplementary Text
Figs. S1 to S15
Tables S1 to S3
References

The Supplementary Materials (SM) is organized as follows: First, we discuss code and data availability (SM A), including links to multiple repositories to reproduce the different ML-based closures and climate simulations discussed in the manuscript. Then, we present the characteristics of the emulated mapping (SM B1), derive the input transformations used in the manuscript (SM B2), the output transformations tested in this SM (SM B3), and discuss possible vertical coordinate transformations (SM B4). We provide a guide to find new climate-invariant transformations in SM B5. SM C details the practical implementation of our climate-invariant ML workflow. Finally, we present supplementary results in SM D, including the Hellinger and Jensen-Shannon distances between input distributions (SM D1), the learning curves of climate-invariant models across climates and geographies (SM D2), the generalization skill of climate-invariant NNs near the surface (SM D3), and three methods to visualize the “raw-data” and climate-invariant mappings and compare them in the cold (-4K) and warm (+4K) climates (SM D4).

# A. Code and Data Availability

The code used to process data, train models, and produce this manuscript’s figure can be found in the following Github repository: `https://github.com/tbeucler/CBRAIN-CAM`, which is archived using Zenodo `https://zenodo.org/record/8140413` [97]. This

repository includes a minimal reproducible example on how to train a climate-invariant neural network and verify its improved generalization ability: https://colab.research.google.com/github/tbeucler/CBRAIN-CAM/blob/master/Climate_Invariant_Guide.ipynb and a notebook to generate all figures in this manuscript requiring model data https://github.com/tbeucler/CBRAIN-CAM/blob/master/notebooks/tbeucler_devlog/090_Climate_Invariant_Paper_Figures_v2.ipynb. Both scripts rely on the manuscript's accompanying data, archived in the following Zenodo repository: https://doi.org/10.5281/zenodo.8140536 [98].

The above Github repository is forked from (and builds upon) Stephan Rasp's CBRAIN repository https://github.com/raspstephan/CBRAIN-CAM, also archived using Zenodo https://zenodo.org/record/1402384#.YajSg9BKiUk [99]. This repository contains a quickstart guide https://github.com/raspstephan/CBRAIN-CAM/blob/master/quickstart.ipynb to preprocess raw climate model output, train a neural network and benchmark it.

As described in Section 2, we use data from eight climate simulations using three climate models (SPCAM3, SPCESM2, and SAM) to form our training, validation, and test sets. We report the exact characteristics of the splits in Tab S2 and information to re-generate the full simulation output below.

**SPCAM3** The codebase for running the "SPCAM3" simulation is the same employed by [29], which is archived at https://gitlab.com/mspritch/spcam3.0-neural-net/-/tree/sp-diagnostic for the (+0K) simulation. The sea surface temperature is uniformly cooled by 4K to produce the (-4K) simulation and uniformly warmed by 4K to produce the (+4K) simulation. Raw output of the (+0K) simulation can be found at https://zenodo.org/record/1402384#.YaUCsdDMI-w [99]. The full simulations output, which is several TB, is archived on the GreenPlanet cluster at UC Irvine and available upon request.

**SPCESM2** The codebase for running the "SPCESM2" simulations is the same employed by [100], which is archived at https://github.com/mspritch/UltraCAM-spcam2_0_cesm1_1_1; this code was in turn forked from a development version of the CESM1.1.1 located on the NCAR central subversion repository under tag spcam_cam5_2_00_forCESM1_1_1Rel_V09, which dates to February 25, 2013. The full simulations output, which is several TB, is also archived on the GreenPlanet cluster at UC Irvine and available upon request. We additionally archived the input data and run scripts necesary to re-run all three simulations as part of the manuscript's accompanying data using Zenodo https://zenodo.org/record/5775541#.YbeMHNDMKUl [98].

**SAM** The codebase for running the "SAM" simulations is the same employed by [54]. The initial sounding, meridional surface temperature profile, and source code to re-run the sim-

ulation can be found at `https://zenodo.org/record/4118346#.YaT_WtBKg-w` [101]. To produce the (+0K) simulation, the initial sounding and surface temperature profiles are both uniformly warmed by 4K. The output from the SAM simulations, which is several TB, is archived at MIT and is available upon request.

# B. Feature Transformations

## B1. Mapping

In this subsection, we present the characteristics of the emulated mapping, highlighting the differences between the superparameterized models (SPCAM3, SPCESM2) and the storm-resolving model (SAM). In both cases, the input vector $\boldsymbol{x}$ encodes the large-scale (≈100km) climate state:

$$\boldsymbol{x} = \begin{cases} \begin{bmatrix} \boldsymbol{q}(\boldsymbol{p}) & \boldsymbol{T}(\boldsymbol{p}) & p_s & S_0 & \mathrm{SHF} & \mathrm{LHF} \end{bmatrix}^T & (\mathrm{SPCAM3, SPCESM2}) \\ \begin{bmatrix} \boldsymbol{q}_{\mathbf{np}}(\boldsymbol{p}) & \boldsymbol{T}(\boldsymbol{p}) & d_{\mathrm{Equator}} \end{bmatrix}^T & (\mathrm{SAM}) \end{cases} \quad (1)$$

where $\boldsymbol{q}(\boldsymbol{p})$ is the vertical profile of specific humidity in units of kg/kg (written as a function of the background pressure coordinate $\boldsymbol{p}$ in units of Pa), $\boldsymbol{T}(\boldsymbol{p})$ is the temperature's vertical profile in units of K, $p_s$ is surface pressure in units Pa, $S_0$ is solar insolation in units of $\mathrm{W/m^2}$, SHF is surface sensible heat flux in units $\mathrm{W/m^2}$, LHF is surface latent heat flux in units of $\mathrm{W/m^2}$, $\boldsymbol{q}_{\mathbf{np}}(\boldsymbol{p})$ is the vertical profile of non-precipitating water concentration in units of kg/kg, and $d_{\mathrm{Equator}}$ is the distance to the Equator, which is used as a proxy for solar insolation in the mapping learned for SAM. The output vector $\boldsymbol{y}$ groups subgrid-scale thermodynamic tendencies:

$$\boldsymbol{y} = \begin{cases} \begin{bmatrix} L_v\dot{\boldsymbol{q}}(\boldsymbol{p}) & c_p\dot{\boldsymbol{T}}(\boldsymbol{p}) & c_p\mathbf{lw}(\boldsymbol{p}) & c_p\mathbf{sw}(\boldsymbol{p}) \end{bmatrix}^T & (\mathrm{SPCAM3, SPCESM2}) \\ \begin{bmatrix} L_v\dot{\boldsymbol{q}}_{\mathbf{np}}(\boldsymbol{p}) & \dot{\boldsymbol{H}}_{\boldsymbol{L}}(\boldsymbol{p}) \end{bmatrix}^T & (\mathrm{SAM}) \end{cases} \quad (2)$$

where $L_v$ in units of $\mathrm{J\ kg^{-1}}$ is the latent heat of vaporization of water in standard conditions, $\dot{\boldsymbol{q}}(\boldsymbol{p})$ is the subgrid moistening vertical profile, $c_p$ in units of $\mathrm{J\ kg^{-1}\,K^{-1}}$ is the specific heat of dry air at constant pressure in standard atmospheric conditions, $\dot{\boldsymbol{T}}(\boldsymbol{p})$ is the total subgrid heating vertical profile (including subgrid radiation), $\mathbf{lw}(\boldsymbol{p})$ is the subgrid longwave radiative heating vertical profile, $\mathbf{sw}(\boldsymbol{p})$ is the subgrid shortwave radiative heating vertical profile, $\dot{\boldsymbol{q}}_{\mathbf{np}}(\boldsymbol{p})$ is the non-precipitating water condensation vertical profile, and $\dot{\boldsymbol{H}}_{\boldsymbol{L}}(\boldsymbol{p})$ is the subgrid time-tendency of the liquid/ice static energy vertical profile. Following [60], all components of the output vector $\boldsymbol{y}$ are mass-weighted and vertically integrated within each vertical layer to yield energy flux units ($\mathrm{W/m^2}$). Assuming vertical profiles have $N_p$ vertical levels, $\boldsymbol{x}$ is of length $(2N_p + 4)$ for SPCAM3 and SPCESM2, and of length $(2N_p + 1)$ for SAM. $\boldsymbol{y}$ is of length $4N_p$ for SPCAM3 and SPCESM2, and of length $2N_p$ for SAM.

## B2. Physically-Based Input Transformations

In Fig S2, we compare three transformation options for each input, whose univariate PDFs are depicted in Fig S2: No transformation (top), our most successful transformation (bottom), and our second best transformation (middle). After defining our second best input transformations (B2a), we delve into the details of our relative humidity (B2b) and plume buoyancy (B2c) transformations before discussing the other inputs' distribution shift (B2d).

### B2a. Second Best Input Transformations

Along the way to our optimal feature transformations, we explored candidate options that proved second best. For completeness these are reviewed first in the SM.

**Saturation Deficit:** We explored saturation deficit but found it did not lead to climate invariance. Similar to $\boldsymbol{q}$, saturation deficit (Fig S2a, middle) still has a corresponding expansion of the PDF with warming as a result of the Clausius-Clapeyron relation. It is defined as the amount by which the water vapor concentration must be increased to achieve saturation without changing the environmental temperature and pressure:

$$\tilde{\boldsymbol{q}}_{\mathbf{deficit}}\left(\boldsymbol{T},\boldsymbol{p}\right) \overset{\mathrm{def}}{=} \boldsymbol{q}_{\mathbf{sat}}\left(\boldsymbol{T},\boldsymbol{p}\right) - \boldsymbol{q}, \tag{3}$$

where $\boldsymbol{q}_{\mathbf{sat}}\left(\boldsymbol{T},\boldsymbol{p}\right)$ is the saturation specific humidity.

In contrast, the relative humidity transformation $\tilde{\boldsymbol{q}}_{\mathbf{RH}}\left(\boldsymbol{p}\right) \overset{\mathrm{def}}{=} \mathrm{RH}\left(\boldsymbol{q},\boldsymbol{T},\boldsymbol{p}\right)$ (Fig S2a, bottom) results in a climate-invariant PDF, as evidenced by PDFs that mostly overlap across all three climates.

**Temperature minus Near-Surface Temperature:** Assuming that the temperature's PDF shift with warming is almost uniform with height, we can derive an approximate invariant by subtracting the temperature at all levels $\boldsymbol{T}\left(\boldsymbol{p}\right)$ from the near-surface temperature $T\left(p_{\mathrm{NS}}\right)$:

$$\tilde{\boldsymbol{T}}_{\mathbf{from\ NS}}\left(\boldsymbol{p}\right) \overset{\mathrm{def}}{=} T\left(p_{\mathrm{NS}}\right) - \boldsymbol{T}\left(\boldsymbol{p}\right), \tag{4}$$

where $p_{\mathrm{NS}}$ is the lowest atmospheric pressure level see (Fig S2b, middle). However, this linear transformation fails in the upper atmosphere, especially near the tropopause where temperatures are approximately invariant with warming [78, 79, 80] and therefore decoupled from surface temperature changes. This is why the buoyancy of a moist static energy-conserving plume:

$$\tilde{\boldsymbol{T}}_{\mathbf{buoyancy}}\left(\boldsymbol{p}\right) \overset{\mathrm{def}}{=} \boldsymbol{B}_{\mathbf{plume}}\left(q_{\mathrm{NS}},\boldsymbol{T},\boldsymbol{p}\right), \tag{5}$$

where $q_{\mathrm{NS}} = q(p_{\mathrm{NS}})$ is the near-surface specific humidity, yields approximate climate invariance (Fig S2b, bottom), unlike the temperature minus near surface temperature transformation.

**Scaling Latent Heat Fluxes by Near-Surface Specific Humidity:** To address the increase of LHF with warming, we scale LHF by near-surface specific humidity $q\left(p_{\mathrm{NS}}\right)$ (Fig S2c, middle):

$$\tilde{\mathrm{LHF}}_q \overset{\mathrm{def}}{=} \frac{\mathrm{LHF}}{L_v \max\left\{\epsilon_q, q\left(p_{\mathrm{NS}}\right)\right\}}, \tag{6}$$

where $\epsilon_q$ is a user-chosen parameter that we set to $10^{-4}$ to avoid division by zero. While better than directly using LHF, this transformation fails for very dry atmospheres when the latent heat flux is negative, e.g. in polar oceans where atmospheric water vapor may be condensing on the surface, or when the near-surface specific humidity is very small, e.g. in subtropical regions. This is why scaling LHF using the near-surface saturation deficit (Fig S2c, bottom):

$$\tilde{\mathrm{LHF}}_{\Delta q} \overset{\mathrm{def}}{=} \frac{\mathrm{LHF}}{L_v \max\left\{\epsilon_q, q_{\mathrm{sat}}\left[T\left(p_{\mathrm{NS}}\right), p_{\mathrm{NS}}\right] - q\left(p_{\mathrm{NS}}\right)\right\}}. \tag{7}$$

yields better generalizability. While both the Jensen-Shannon and Hellinger distances would suggest that $\tilde{\mathrm{LHF}}_{\Delta q}$ is a slightly less good transformation than $\tilde{\mathrm{LHF}}_q$, the $\tilde{\mathrm{LHF}}_{\Delta q}$ transformation leads to improved generalization performance compared to $\tilde{\mathrm{LHF}}_q$ (not shown). This confirms that only considering the PDF distances is not always sufficient to find the optimal transformations (discussed in SM B4).

### B2b. Relative Humidity

Relative humidity (RH) provides our optimal transformation for the specific humidity inputs. RH is defined as the ratio of the partial pressure of water vapor $\boldsymbol{e}(\boldsymbol{p}, \boldsymbol{q})$ to its saturation value $\boldsymbol{e}_{\mathbf{sat}}\left(\boldsymbol{T}\right)$, and can be expressed analytically:

$$\mathbf{RH} \overset{\mathrm{def}}{=} \frac{\boldsymbol{e}(\boldsymbol{p}, \boldsymbol{q})}{\boldsymbol{e}_{\mathbf{sat}}\left(\boldsymbol{T}\right)} \overset{q \ll 1}{\approx} \frac{R_v}{R_d} \frac{\boldsymbol{pq}}{\boldsymbol{e}_{\mathbf{sat}}\left(\boldsymbol{T}\right)}, \tag{8}$$

where $R_v \approx 461\mathrm{J\ kg^{-1}\ K^{-1}}$ is the specific gas constant for water vapor, $R_d \approx 287\mathrm{J\ kg^{-1}\ K^{-1}}$ is the specific gas constant for dry air, $\boldsymbol{p}$ (in units Pa) is the total atmospheric pressure, $\boldsymbol{q}$ (in units kg/kg) is specific humidity, and $\boldsymbol{e}_{\mathbf{sat}}\left(\boldsymbol{T}\right)$ (in units Pa) is the saturation pressure of water vapor, whose analytic expression in our case is given below. Consistent with Eq 8, the saturation specific humidity $\boldsymbol{q}_{\mathbf{sat}}$ corresponding to $\mathrm{RH} = 1$, is

$$\boldsymbol{q}_{\mathbf{sat}}\left(\boldsymbol{T}, \boldsymbol{p}\right) = \frac{R_d \boldsymbol{e}_{\mathbf{sat}}\left(\boldsymbol{T}\right)}{R_v \boldsymbol{p}}. \tag{9}$$

SAM's single-moment microphysics scheme [102], which is also used in the SPCAM3 and SPCESM2 simulations, partitions water between the liquid and ice phases using a weight $\omega$ that is a linear function of the absolute temperature:

$$\boldsymbol{\omega} \overset{\mathrm{def}}{=} \frac{\boldsymbol{T} - T_{00}}{T_0 - T_{00}}. \tag{10}$$

Under the assumptions of this microphysics scheme, the saturation pressure of water vapor can then be found by integrating the Clausius-Clapeyron equation with respect to temperature, expressed analytically as:

$$\boldsymbol{e}_{\mathbf{sat}}(\boldsymbol{T}) = \begin{cases} \boldsymbol{e}_{\mathbf{liq}}(\boldsymbol{T}) & \boldsymbol{T} > T_0 \\ \boldsymbol{e}_{\mathbf{ice}}(\boldsymbol{T}) & \boldsymbol{T} < T_{00} \\ \boldsymbol{\omega}\boldsymbol{e}_{\mathbf{liq}}(\boldsymbol{T}) + (\mathbf{1} - \boldsymbol{\omega})\,\boldsymbol{e}_{\mathbf{ice}}(\boldsymbol{T}) & \boldsymbol{T} \in [T_{00}, T_0] \end{cases}, \tag{11}$$

where $T_0 = 273.16\mathrm{K}$ and $T_{00} = 253.16\mathrm{K}$. In Eq 11, as temperature increases, the saturation pressure of water vapor goes from the saturation vapor pressure with respect to liquid $\boldsymbol{e}_{\mathbf{liq}}$, to the saturation vapor pressure with respect to ice $\boldsymbol{e}_{\mathbf{ice}}$. These are given by the following polynomial approximations:

$$\boldsymbol{e}_{\mathbf{liq}}(\boldsymbol{T}) = 100\mathrm{Pa} \times \sum_{i=0}^{8} a_{\mathrm{liq,i}} \left[\max\left(-193.15\mathrm{K}, T - T_0\right)\right]^i, \tag{12}$$

where $\boldsymbol{a}_{\mathbf{liq}}$ is a vector of length 9 containing nonzero polynomial coefficients. The polynomial approximation for $\boldsymbol{e}_{\mathbf{ice}}$, with the same temperature switches as Eq 11, is:

$$\boldsymbol{e}_{\mathbf{ice}}(\boldsymbol{T}) = \begin{cases} \boldsymbol{e}_{\mathbf{liq}}(\boldsymbol{T}) \\ 100\mathrm{Pa} \times \left\{c_{\mathrm{ice,1}} + \boldsymbol{\mathcal{C}}(\boldsymbol{T})\left[c_{\mathrm{ice,4}} + c_{\mathrm{ice,5}}\boldsymbol{\mathcal{C}}(\boldsymbol{T})\right]\right\} \\ 100\mathrm{Pa} \times \sum_{i=0}^{8} a_{\mathrm{ice,i}} \left(\boldsymbol{T} - T_0\right)^i \end{cases}, \tag{13}$$

where $\boldsymbol{\mathcal{C}}(\boldsymbol{T})$ is a ramp function of temperature given by:

$$\boldsymbol{\mathcal{C}}(\boldsymbol{T}) \overset{\mathrm{def}}{=} \max\left(c_{\mathrm{ice,2}}, \boldsymbol{T} - T_0\right), \tag{14}$$

and $(\boldsymbol{a}_{\mathbf{ice}}, \boldsymbol{c}_{\mathbf{ice}})$ are vectors of length 9 and 5 containing nonzero elements, respectively. Between temperatures of $T_{00}$ and $T_0$, the saturation pressure of water vapor is a weighted mean of $\boldsymbol{e}_{\mathbf{liq}}$ and $\boldsymbol{e}_{\mathbf{ice}}$. The reader interested in the numerical details of this transformation is referred to our implementation of relative humidity at `https://colab.research.google.com/github/tbeucler/CBRAIN-CAM/blob/master/Climate_Invariant_Guide.ipynb`.

**B2c. Plume Buoyancy**

Plume Buoyancy ($B_{\mathrm{plume}}$) is our most successful transformation for the temperature inputs. Buoyancy is defined as the upward acceleration exerted upon parcels by virtue of the density difference between the parcel and the surrounding air of the atmospheric column (e.g., [103]). Because our ML model's inputs represent the large-scale thermodynamic state, the ML model does not have information about the storm-scale buoyancy field, and we must rely on idealized approximations to estimate the buoyancy that a plume would have for given specific humidity and temperature profiles. To be consistent with the model's conserved quantities [102], we

derive a simple buoyancy metric based on a moist static energy ($h$) conserving plume below following similar derivations in [82] and [104]. We refer the reader interested in the numerical details of this transformation to https://colab.research.google.com/github/tbeucler/CBRAIN-CAM/blob/master/Climate_Invariant_Guide.ipynb.

For purposes of this transformation, we omit virtual temperature effects and condensate loading (effects of the environmental water vapor on heating/moisture sink are being estimated separately). Thus the parcel buoyancy is simply proportional to the relative difference between its temperature $T_{\mathrm{par}}$ and the environmental temperature $T$:

$$\boldsymbol{B}_{\mathbf{plume}} \approx g \frac{T_{\mathrm{par}} - \boldsymbol{T}}{\boldsymbol{T}}, \tag{15}$$

where $g$ is the gravity constant. Further assuming that the plume is non-entraining, obeys hydrostatic balance, and lifts parcels from the near-surface, the lifted parcel's moist static energy is conserved and equal to its near-surface value (at pressure $p_{\mathrm{NS}}$:

$$\begin{aligned} h_{\mathrm{par}} &\approx h\left(p_{\mathrm{NS}}\right) \\ &\overset{\mathrm{def}}{=} L_v q\left(p_{\mathrm{NS}}\right) + c_p T\left(p_{\mathrm{NS}}\right), \end{aligned} \tag{16}$$

where we have used the environmental moist static energy's definition:

$$\boldsymbol{h} \overset{\mathrm{def}}{=} L_v \boldsymbol{q} + c_p \boldsymbol{T} + g\boldsymbol{z}, \tag{17}$$

where $\boldsymbol{z}$ is geopotential height, and we neglected $z\left(p_{\mathrm{NS}}\right)$ as the near-surface is close to the surface by definition. To express the parcel's buoyancy as a function of the environmental thermodynamic state, we finally assume that the parcel is saturated (not necessarily true close to the surface), and that the thermodynamic differences between the parcel and the environment are small, which allows us to linearize the Clausius-Clapeyron equation about the environmental temperature:

$$\begin{aligned} T_{\mathrm{par}} - \boldsymbol{T} &\overset{\mathrm{Claus.-Clap.}}{\approx} \left(\frac{\partial T}{\partial q^*}\right)_{\boldsymbol{T},\boldsymbol{p}} \left(q_{\mathrm{sat,par}} - \boldsymbol{q}_{\mathbf{sat}}\right) \\ &= \frac{R_v \boldsymbol{T}^2}{L_v \boldsymbol{q}_{\mathbf{sat}}} \left(q_{\mathrm{sat,par}} - \boldsymbol{q}_{\mathbf{sat}}\right) \\ &= \frac{R_v \boldsymbol{T}^2}{L_v^2 \boldsymbol{q}_{\mathbf{sat}}} \left[h_{\mathrm{par}} - \boldsymbol{h}_{\mathbf{sat}} - c_p\left(T_{\mathrm{par}} - \boldsymbol{T}\right)\right]. \end{aligned} \tag{18}$$

Using Eq 18 to write $T_{\mathrm{par}} - \boldsymbol{T}$ as a function of the environmental thermodynamic state and substituting the resulting expression into Eq 15 yields an estimation of plume buoyancy from $(\boldsymbol{q}, \boldsymbol{T}, \boldsymbol{p})$:

$$\boldsymbol{B}_{\mathbf{plume}}(\boldsymbol{q}, \boldsymbol{T}, \boldsymbol{p}) = \frac{g\left[h_{\mathrm{par}} - \boldsymbol{h}_{\mathbf{sat}}\left(\boldsymbol{q}, \boldsymbol{T}, \boldsymbol{p}\right)\right]}{\boldsymbol{\kappa}\left(\boldsymbol{T}, \boldsymbol{p}\right) \times c_p \boldsymbol{T}}, \tag{19}$$

where the parcel's moist static energy is expressed as a function of near-surface $(q, T)$ in Eq 16, the environmental saturated moist static energy in pressure coordinates is defined as:

$$\boldsymbol{h}_{\mathbf{sat}}\left(\boldsymbol{q}, \boldsymbol{T}, \boldsymbol{p}\right) \stackrel{\text{def}}{=} L_v \boldsymbol{q}_{\mathbf{sat}}\left(\boldsymbol{T}, \boldsymbol{p}\right) + c_p \boldsymbol{T} + g \boldsymbol{z}\left(\boldsymbol{q}, \boldsymbol{T}, \boldsymbol{p}\right), \tag{20}$$

and we have introduced the dimensionless factor:

$$\boldsymbol{\kappa}\left(\boldsymbol{T}, \boldsymbol{p}\right) = 1 + \frac{L_v^2 \boldsymbol{q}_{\mathbf{sat}}\left(\boldsymbol{T}, \boldsymbol{p}\right)}{R_v c_p \boldsymbol{T}^2}. \tag{21}$$

Note that in pressure coordinates, we calculate the geopotential height by vertically integrating the hydrostatic equation after using the ideal gas law:

$$\boldsymbol{z}\left(\boldsymbol{q}, \boldsymbol{T}, \boldsymbol{p}\right) = \int_p^{p_{\mathrm{NS}}} dp' \frac{T\left(p'\right)}{gp'} \left\{R_d + \left[R_v - R_d\right] q\left(p'\right)\right\}. \tag{22}$$

#### B2d. Sensible Heat Fluxes and Surface Pressure

In this appendix, we discuss the univariate PDFs of the two inputs we did *not* transform in the main manuscript (see Section 3) in Fig S3 for both super-parameterized models and all three surface temperatures (-4K, +0K, and +4K). The PDF of sensible heat fluxes changes very little with warming. There is a slight expansion of the left tail of the surface pressure PDF with warming as the most extreme low-pressure systems become more intense, but we hypothesize that these changes are small enough not to require a dedicated input transformation.

## B3. Output Transformation

#### B3a. Theory

In contrast to input transformation, transforming our ML models' outputs, namely subgrid thermodynamics, only marginally improves the models' ability to generalize. In the absence of physical theory on how the full vertical profile of subgrid thermodynamics changes with warming, we place ourselves in an idealized scenario:

Assuming we know how the outputs' marginal PDF changes with warming, can we help our ML models generalize via output transformation?

We note that assuming knowledge of how the *marginal* (univariate) PDFs (or equivalently the CDF) of convective heating and moistening change with warming is more realistic than assuming full knowledge of how their *joint* PDFs change with warming. This knowledge could come from e.g. convection theory (e.g., [73]) or shorter simulations than those required to train a subgrid closure. Under this assumption, a natural transformation is the outputs' cumulative distribution function (CDF):

$$\tilde{\boldsymbol{y}} = \mathrm{CDF}\left(\boldsymbol{y}\right). \tag{23}$$

In essence, we are assuming that the mapping is more likely to be invariant in quantile than in physical space, which is a common practice when debiasing the outputs of climate models referred to as *quantile mapping* (e.g., review by [105]). In practice, we test two distinct methods for transforming the outputs using their CDFs and report the results for SPCAM3 aquaplanet simulations in SM B2b.

**Quantile mapping after training:** The first method is to transform the ML model's input *during* training, and then transform the ML model's output *after* training. This is akin to standard, post-hoc, quantile mapping. In the particular case of trying to generalize from a (-4K) cold simulation to a (+4K) warm simulation, the entire transformation to yield outputs in physical units can be mathematically written as:

$$y \mapsto \mathrm{CDF}_{+4\mathrm{K}}^{-1}\left[\mathrm{CDF}_{-4\mathrm{K}}\left(y\right)\right], \tag{24}$$

where for simplicity but without loss of generality, we have considered a singular input $y$ whose CDF is $\mathrm{CDF}_{-4\mathrm{K}}$ in the (-4K) cold simulation and $\mathrm{CDF}_{+4\mathrm{K}}$ in the (+4K) warm simulation.

**Quantile mapping before training:** The second method is to transform the ML model's output *before* training. In that case, we directly train the ML model to predict $\tilde{y} = \mathrm{CDF}\left(y\right)$ as accurately as possible. We then map the output back to physical units using $\mathrm{CDF}^{-1}$ *after* training.

### B3b. Results

The two methods to transform outputs presented above are depicted in Fig S4 and the generalization results presented in Fig S5. Transforming outputs *after* training slightly improves generalization skill (from an overall $R^2$ of 0.58 to 0.62 for the generalization (+4K) set). In contrast, transforming outputs *before* training leads to equally bad results both on the training and generalization sets, which is a negative result underlining the challenges of designing the appropriate loss function in probability space. A possible solution would be to convert back the outputs to physical space before feeding them to the loss function during training, and further investigation is required to fully assess the potential and limitations of training these ML models in probability space.

## B4. Spatial Coordinate Transformation

Another transformation to consider when input/output variables are functions of spatiotemporal coordinates is *coordinate transformation*, resulting in a coordinate change. In our specific example, it is possible to transform the vertical coordinate, i.e. the hybrid pressure coordinate $p$. Possible transformations include:

1. the temperature ($\widetilde{p} = T$), e.g. for radiative heating, which tends to vary less in temperature coordinates [106],

2. the saturation specific humidity ($\widetilde{p} = q_{\mathrm{sat}}$) which is consistent with a transformation of the primitive equations that captures an upward shift of the circulation as the climate warms [107],

3. the geopotential height or the altitude ($\widetilde{p} = z$), which could more consistently capture gravity wave propagation,

4. or a coordinate with fixed values for characteristic vertical levels in the atmosphere, such as the top of the planetary boundary layer or the tropopause [78].

While we were able to transform the vertical coordinate using interpolation functions (not shown), the benefits were not visible in our particular c ase. This could be because input transformation already addresses some of the upward shift of convective activity warming, as shown by the influence of humidity inputs in Fig S14.

# C. Implementation

For reproducibility purposes [108], we now detail the practical implementation of a climate-invariant ML workflow (see Fig 9), from its overall structure (SM C1) to its benchmarking (SM C4) via the characteristics of the multiple linear regressions (MLR, SM C2) and neural networks (NN, SM C3) presented in this manuscript.

## C1. Overall Workflow

We present three ways to implement physical transformations. The first w ay i s t o physically transform the inputs/outputs *before* training. While this option is easiest to implement and debug, it usually comes at the cost of disk space: Every time we try a new transformation, we need to duplicate our training/validation/test datasets for all the climates/geographies we are interested in, which can quickly be prohibitive when trying multiple transformation combinations.

Therefore, it can be advantageous to transform the input/output variables within the ML framework, so that the transformations occur *during* training. In essence, we are trading disk space for computational time. In that spirit, the second method is to transform the inputs/outputs via custom layers (e.g., Ch 12 of [70]) in the ML algorithm itself. Since this second method tends to substantially slow down training as it adds sequential operations on the GPU, we take advantage of the fact that the transformations occur before and after the emulated mapping, and propose a third method that can happel in parallel on the CPU: Transforming inputs/outputs by customizing the pipeline or "data generator", which is the algorithm responsible for feeding numbers to the ML model after reading the training data files. For each batch, the *custom data*

*generator* then transforms inputs *before* feeding them to the ML algorithm. In our case, note that we transform outputs independently via quantile mapping (see SM B2).

For the rest of this manuscript, we will train our ML models using custom data generators: For "raw-data" models, the transformations are set to None (no transformation), while for "climate-invariant" models, the $q$ transformation is set to $\tilde{q}_{\mathrm{RH}}$, the $T$ transformation is set to $\tilde{T}_{\mathrm{buoyancy}}$, and the LHF transformation is set to $\widetilde{\mathrm{LHF}}_{\Delta q}$. For all models, we additionally subtract the mean from each input before dividing it by its range to feed the ML algorithm floating-point numbers between (-1) and 1. Note that for each transformation, numbers are "de-normalized" before the transformation and "re-normalized" after following the normalization procedure described in Sec 2.4. Therefore, all transformations are done in physical units while the ML algorithm is always fed single-precision floating-point numbers in $[-1, 1]$.

For simplicity and building upon previous ML-powered subgrid closures [29, 64, 54], we use the mean-squared error (MSE) of the prediction *in physical units* (here $\mathrm{W}^2\mathrm{m}^{-4}$) as our loss function. Motivated by the framework presented in Fig 9, we first train MLRs (SM C2) before training NNs (SM C3) and benchmarking our ML models to quantify their accuracy and ability to generalize (SM C4).

## C2. Multiple Linear Regressions

To use the same data generator for both MLRs and NNs, we implement our MLRs in Tensorflow 2.0 [109] and train them using the Adam optimizer, which builds on stochastic gradient descent [110]. Training a climate-invariant MLR results in a weight matrix $\boldsymbol{A}$ of size $4N_p \times (2N_p + 4)$ and a bias vector $\boldsymbol{b}$ of length $4N_p$ such that:

$$\boldsymbol{y} \approx \boldsymbol{A}\tilde{\boldsymbol{x}} + \boldsymbol{b}, \tag{25}$$

where stochastic optimization means that there is no unique optimal solution for $\boldsymbol{A}$ and $\boldsymbol{b}$. We train MLRs for 20 epochs using the default Keras learning rate of 0.001 and save the weights and biases corresponding to the minimal loss over the validation set.

## C3. Neural Network Design

To isolate the effects of physically transforming the NN's inputs, we fix the hyperparameters of all NNs trained in this study, and leave the joint investigation of hyperparameter tuning and physical transformations for future work. Informed by [50] and [52], we fix the architecture to a multilayer perceptron of 7 layers of 128 neurons separated by Leaky Rectified Linear Unit activation functions of slope 0.3, resulting in 122,872 trainable parameters for each NN. We implement the SPCAM NNs using Tensorflow 2.0 [109], train them for 20 epochs using the Adam optimizer with the default Keras learning rate of 0.001 and a default batch size of 1024, and save the parameters corresponding to the minimal validation loss.

Following the supplemental material (Sec 2) of [55], some of the hyperparameters used for the NNs trained on SAM data are different. The SAM NNs are implemented using PyTorch

1.4.0 [111], have 5 dense layers of 128 neurons each, and use cyclic learning rate [112]: Starting with an initial learning rate in $[2\ \times 10^{-4}, 2\ \times 10^{-3}]$ for the first epoch out of 10, we then reduce the minimal and maximal learning rates by $10\%$ for the next 6 epochs before further reducing them by a factor 10 for the last 3 epochs.

For SPCAM and following [34], we augment some of our NNs with BN and DP layers, more specifically one DP layer before each activation function and a single BN layer before the first DP layer. Following [37], we use the default DP rate of 30% and the default parameters of the Keras BN layer that normalize each feature using its mean and standard deviation in a given batch [36]. Note that we do not adjust the default parameters of DP and BN to optimize *generalization* skill as this would require misusing the generalization test set as a validation test.

## C4. Benchmarking

We benchmark our ML models using two different metrics: their MSEs and their coefficient of determination $R^2$, defined for a singular output $y_k$ as:

$$R^2 \stackrel{\text{def}}{=} 1 - \frac{\left\langle y_{\text{Err,k}}^2 \right\rangle_{\text{samp}}}{\left\langle \left( y_{\text{Truth,k}} - \left\langle y_{\text{Truth,k}} \right\rangle_{\text{samp}} \right)^2 \right\rangle_{\text{samp}}}, \quad (26)$$

where $\langle \cdot \rangle_{\text{samp}}$ is the averaging operator over the samples of interest. For instance, if we want a horizontal map of $R^2$, we average samples at a given location over time, while we average over time and horizontal space if we want a single $R^2$ value for $y_k$. Similarly, if we want one value of MSE per output $y_k$, we only average the MSE over time and horizontal space rather than over all outputs, as when calculating the loss function.

While comparing MSE and $R^2$ in the reference and target generalization climates is enough to assess generalization skill *after* training, we are also interested in how a given ML model learns to generalize *during* training. To address that question, we augment our SPCAM ML models with a function (technically a "Keras callback" [113]) that calculates the MSE over two datasets that correspond to the two generalization experiments at the end of each epoch: (1) a dataset of different temperature (warm when training on cold, and vice-versa); and (2) a dataset of different geography (Earth-like when training on Aquaplanet, and vice-versa). At the end of training, we hence obtain three learning curves for each ML model: the validation loss, and the loss in the two generalization sets as a function of number of epochs. Note that these callbacks are computationally expensive as they require evaluating the ML model over $\approx 100M$ samples at the end of each epoch, which means they should be avoided when purely seeking performance, e.g. during hyperparameter tuning.

# D. Supplementary Results

## D1. Jensen-Shannon Distance between PDFs across Climates

As an alternative to the Hellinger PDF distance, we pick the Jensen-Shannon distance [96] because it is a symmetric distance (i.e., the arguments' order does not affect the outcome) that uses the logarithms of the PDFs, hence giving large weights to the PDFs' tails that tend to be particularly problematic for generalization purposes:

$$\mathrm{JS}\left(p,q\right)\overset{\mathrm{def}}{=}\sqrt{\frac{\mathrm{KL}\left(p,q\right)+\mathrm{KL}\left(q,p\right)}{2}},\tag{27}$$

where $p$ and $q$ are the normalized PDFs to compare and KL is the Kullback–Leibler divergence, defined for continuous PDFs as:

$$\mathrm{KL}\left(p,q\right)\overset{\mathrm{def}}{=}\int_{0}^{1}dx\times p\left(x\right)\ln\left[\frac{p\left(x\right)}{q\left(x\right)}\right].\tag{28}$$

## D2. Learning across Climates and Geographies

This section complements Sec 4.2 and confirms that climate-invariant models learn mappings that are valid across climates and geographies *during* training. For this purpose, we track the models' generalizability throughout the training process as explained below.

Fig S7 shows learning curves; the color of each line indicates the dataset the model was *trained* in, while the color of the row indicates the dataset the model was *tested* in. To gain intuition, we can start by looking at lines that have the same color as their axes: These are the "standard" learning curve showing that each model's validation loss in the same climate/geography monotonically decreases as the model is trained, confirming that we are not overfitting the training set.

We are now ready to zoom in on a key result of this manuscript: The learning curve of the "climate-invariant" NN trained in the cold aquaplanet but tested in the warm aquaplanet (starred blue line in the red box (a)). Impressively, this learning curve is mostly decreasing, confirming that *"climate-invariant" NNs are able to continuously learn about subgrid thermodynamics in the warm aquaplanet as they are trained in the cold aquaplanet.* In contrast, the "raw-data" NN trained in the cold aquaplanet but tested in the warm aquaplanet (circled blue line in the red box (a)) makes extremely large generalization errors, which worsen as the model is trained in the cold aquaplanet.

*"Climate-invariant" NNs also facilitate learning across geographies*, i.e., from the aquaplanet to the Earth-like simulations (starred blue line in green box (b) is consistently below circled blue line) and vice-versa (starred green line in blue box (c) is consistently below circled green line). "Climate-invariant" transformations additionally improve the MLR baseline's generalization ability (see right column, e.g., starred blue line in red box (a) and starred green line in

blue box (c)), albeit less dramatically. This smaller improvement in MLR's generalization abilities is linked to its relatively small number of free parameters, resulting in (1) "raw-data" MLRs generalizing better than "raw-data" NNs; and (2) MLRs having lower representation power and fitting their training sets less well, limiting the maximal accuracy of "climate-invariant" MLRs on the test set.

There are a few cases in which transforming inputs does not fully solve the generalization problem, e.g., when trying to generalize from the aquaplanet to the Earth-like simulation (starred blue line in green box (b)). NNs with DP fit their training set less well (squared lines that have the same color as their boxes are above corresponding circled/starred lines). However, they improve generalization in difficult cases (e.g., squared blue line in green box (b)) and do not overly deteriorate generalization in cases where the input transformations work particularly well (e.g., squared green line in blue box (a)). This confirms that *combining physics-guided generalization methods (e.g., physical transformation of the inputs/outputs) with standard ML generalization methods (e.g., DP)* is advantageous.

## D3. Geographic Skill

This section complements Sec 4.3 by presenting different cross-sections of NN skill *after* training. Our results confirm that while raw-data NN trained in the cold climate struggle to generalize to the warm climate's Tropics, the climate-invariant mapping alleviates this limitation.

Fig S8a, which shows cross-section of the coefficient of determination $R^2$ (1 or yellow for perfect predictions, and -1 or blue for errors larger or equal to two standard deviations) exposes the raw-data NN's poor generalization skill in the warm (+4K) Tropics. In contrast, Fig S8b underlines how climate-invariant NNs improve generalization throughout the atmosphere in the warm Tropics without deteriorating skill in the mid-latitudes and poles of the warm simulation. This consideration helped us choose our final input transformation, as the $\tilde{\boldsymbol{T}}_{\mathbf{from\ NS}}$ temperature transformation significantly deteriorated generalization in the mid-latitudes, while the $\tilde{\boldsymbol{T}}_{\mathbf{buoyancy}}$ transformation helps generalization in the Tropics without overly compromising skills at other latitudes. There is a slight skill compromise at high latitudes, as can be seen by comparing the second rows of Fig S8a and Fig S8b, which is especially apparent in the SAM case and can be partially traced back to challenges in generalizing subgrid ice sedimentation (not shown here, see [54] for details).

To show that the improved generalization skill of climate-invariant NNs for subgrid heating is not unique to the mid-troposphere (see Fig 5), in Fig S9 we also show the generalization skill of climate-invariant NNs near the surface. Consistent with [52, 23], the highest skill for the training climate is over land for all NNs as most of the variability comes from the diurnal cycle, which is easy to predict for NNs. Similarly to Fig 5, the generalization error is apparent for the raw-data NN (a) and mostly solved by making the NN climate-invariant (b).

## D4. Visualizing Climate-Invariant Mappings

Before using SHAP in Section 4.4 to visualize the difference between raw-data and climate-invariant mappings, we test simple linear methods to analyze ML models. First, we directly plot the weights $\boldsymbol{A}$ (see Eq 25) of our multi-linear regressions in Fig S10/S11. Second, we plot the mean Jacobian of our NN calculated via automatic differentiation in Fig S12/S13. Unlike SHAP, the MLR weights and the Jacobian matrices both suggest that the climate-invariant mapping is non-local in the vertical. Fig S10/S11 is consistent with the climate-invariant MLR generalizing only slightly better than the raw-data MLR (see top-right panel of Fig S7). Meanwhile, comparing Fig S12/S13 to the full SHAP feature importance matrix (Fig S14/S15) suggests that while the linear sensitivity of subgrid heating/moistening with respect to lower-tropospheric plume buoyancy is high (top panels of Fig S12b), which is expected, subgrid heating/moistening can be well-predicted using mostly local plume buoyancy information (top panels of Fig S14b).

| Row | Input | SPCAM3 | SPCESM2 | SAM |
|---|---|---|---|---|
| 1 | $q_{600\text{hPa}}$ | 20.3, 35.1 | 17.1, 29.5 | 22.1 |
| 2 | $q_{\text{deficit},600\text{hPa}}$ | 24.9, 36.5 | 18.1, 31.0 | 30.0 |
| 3 | $\text{RH}_{600\text{hPa}}$ | 3.6, 8.2 | 3.2, 5.3 | 4.3 |
| 4 | $T_{850\text{hPa}}$ | 53.2, 64.3 | 25.7, 37.3 | 51.9 |
| 5 | $T_{\text{from NS},850\text{hPa}}$ | 5.1, 6.2 | 3.3, 6.4 | 10.5 |
| 6 | $B_{\text{plume},850\text{hPa}}$ | 9.4, 14.7 | 3.6, 7.6 | 5.8 |
| 7 | $T_{150\text{hPa}}$ | 30.2, 33.5 | 31.6, 34.4 | 65.6 |
| 8 | $T_{\text{from NS},150\text{hPa}}$ | 38.0, 53.7 | 14.5, 28.7 | 51.0 |
| 9 | $B_{\text{plume},150\text{hPa}}$ | 35.1, 42.2 | 10.4, 20.9 | 21.1 |
| 10 | LHF | 8.6, 14.5 | 9.7, 12.5 | |
| 11 | $\text{LHF}_q$ | 4.7, 9.5 | 10.0, 10.7 | |
| 12 | $\text{LHF}_{\Delta q}$ | 6.3, 9.9 | 9.9, 14.0 | |

Table S1: **Hellinger distance (in %) away from the (-4K) simulation for the PDFs of key inputs** $(q_{600\text{hPa}}, T_{850\text{hPa}}, T_{150\text{hPa}}, \text{LHF})$ **and their transformations.** (+0K) distance in gray and (+4K) distance in red.

| Model | Spatiotemporal Resolution | Training Set | Validation Set | Test Set |
|---|---|---|---|---|
| SPCAM3 | $(2.8°\times2.8°)_{\text{T42}}\times$30lev$\times$30min | Yr2, Mo1-4→47M | Yr2, Mo5-8→48M | Yr1, Mo6-9→48M |
| SPCESM2 | 2.5°×1.9°×30lev×15min | Yr1, Day0-9/Mo→143M | Yr2, Day0-9/Mo→143M | Yr2, Day20-28/Mo →118M |
| SAM (-4K) | 96km×96kmx48lev×180min | day 225-545 → 13.8M | day 545-562 → 0.7M | day 562-587 → 2.6M |
| (+0K) | 96kmx96km×48lev×180min | - | - | day 380-405 → 2.6M |

Table S2: **Characteristics of the training/validation/test sets used in this manuscript.** The spatiotemporal resolution uses the format longitude × latitude × vertical levels × time. For SPCAM3, which uses a T42 spectral truncation, we use months 1 to 4 of the second simulation year to build the training set, resulting in $\approx 47M$ samples. For SPCESM2, we use the first 9 days of every month of the first simulation year to build the training set, resulting in $\approx 143M$ samples.

| Row | Input | SPCAM3 | SPCESM2 | SAM |
|---|---|---|---|---|
| 1 | $q_{600\text{hPa}}$ | 0.5, 0.8 | 0.4, 0.7 | 0.5 |
| 2 | $q_{\text{deficit},600\text{hPa}}$ | 0.7, 1.0 | 0.4, 0.8 | 0.8 |
| 3 | $\text{RH}_{600\text{hPa}}$ | 0.1, 0.2 | 0.1, 0.1 | 0.1 |
| 4 | $T_{850\text{hPa}}$ | 1.4, 1.9 | 0.5, 0.8 | 1.3 |
| 5 | $T_{\text{from NS},850\text{hPa}}$ | 0.1, 0.1 | 0.1, 0.1 | 0.2 |
| 6 | $B_{\text{plume},850\text{hPa}}$ | 0.2, 0.3 | 0.1, 0.2 | 0.1 |
| 7 | $T_{150\text{hPa}}$ | 0.6, 0.7 | 0.7, 0.7 | 1.5 |
| 8 | $T_{\text{from NS},150\text{hPa}}$ | 0.9, 1.4 | 0.3, 0.6 | 1.4 |
| 9 | $B_{\text{plume},150\text{hPa}}$ | 1.0, 1.2 | 0.2, 0.4 | 0.5 |
| 10 | LHF | 0.2, 0.3 | 0.2, 0.3 | |
| 11 | $\text{LHF}_q$ | 0.1, 0.2 | 0.2, 0.2 | |
| 12 | $\text{LHF}_{\Delta q}$ | 0.1, 0.2 | 0.2, 0.3 | |

Table S3: **Jensen-Shannon distance away from the (-4K) simulation for the PDFs of key inputs** $(q_{600\text{hPa}}, T_{850\text{hPa}}, T_{150\text{hPa}}, \text{LHF})$ **and their transformations.** (+0K) distance in gray and (+4K) distance in red.

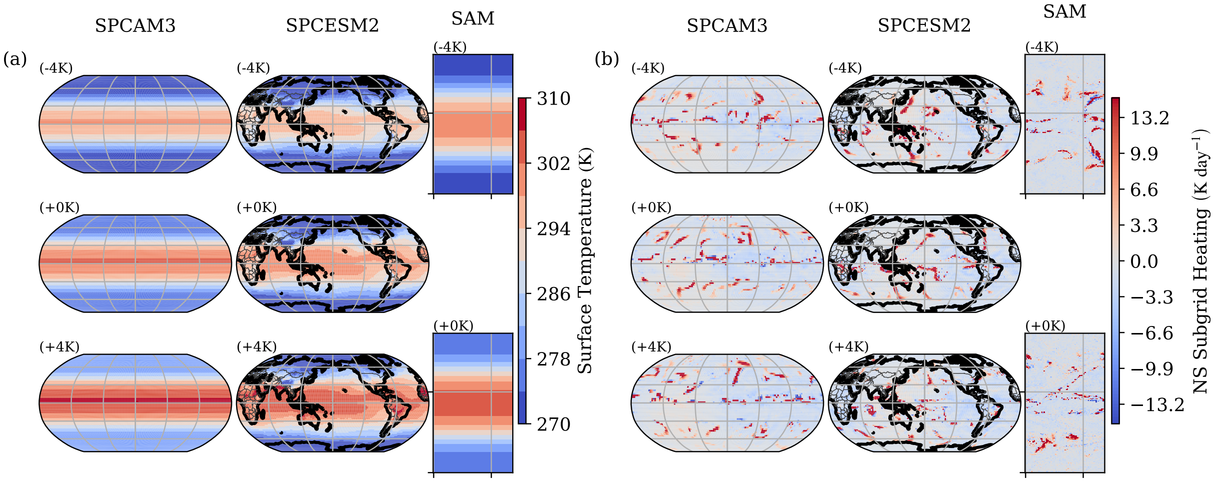


Figure S1: **Surface temperatures and subgrid heating rate in the three utilized atmospheric models.** (a) Prescribed surface temperature (in K) for (left) the aquaplanet SPCAM3 model and (right) the hypohydrostatic SAM model. (center) Annual-mean, near-surface air temperatures in the Earth-like SPCESM2 model. (b) Snapshots of near-surface subgrid heating rate (in K/day). For each model, we show the cold (-4K), reference (+0K), and warm (+4K) simulations.

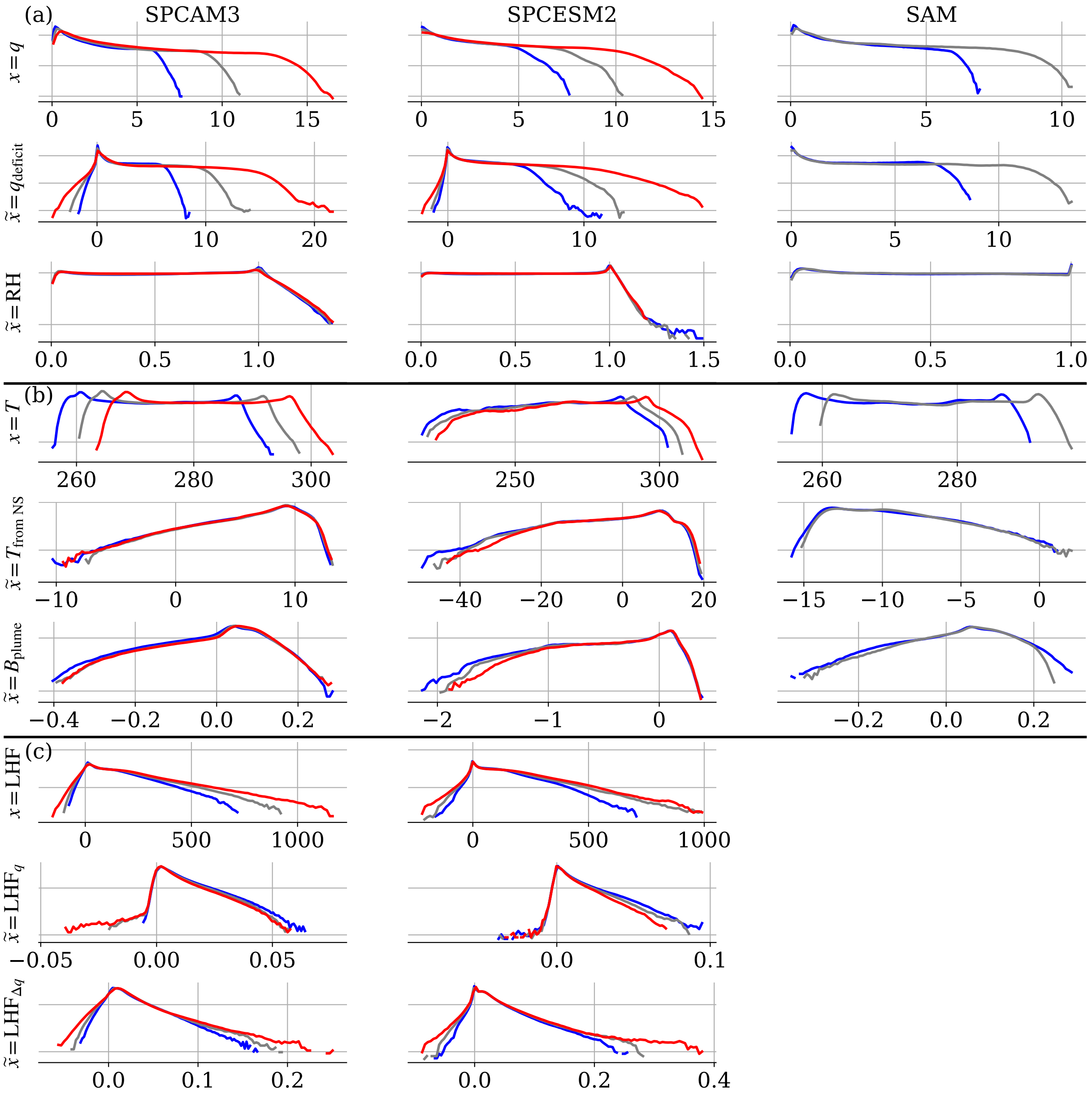


Figure S2: **Univariate PDFs of the (a) 600hPa specific humidity, (b) 850hPa temperature, and (c) latent heat flux in the cold (blue), reference (gray), and warm (red) simulations of each model (SPCAM3, SPCESM2, and SAM).** For each variable, we also show the PDFs of the two transformations discussed in SM SB.2. From top to bottom, the variables are $q$ (g/kg), $q_{\mathrm{deficit}}$ (g/kg), RH, T (K), $T_{\mathrm{from\,NS}}$ (K), $B_{\mathrm{plume}}$ (m/s$^2$), LHF (W/m$^2$), $\mathrm{LHF}_q$ (kg m$^{-2}$s$^{-1}$), and $\mathrm{LHF}_{\Delta q}$ (kg m$^{-2}$s$^{-1}$). For a given variable and transformation, we use the same vertical logarithmic scale across models. Note that unlike for $q$, the best options for $T$ and LHF do not decrease distribution distance more than the second best options, which is discussed in text.

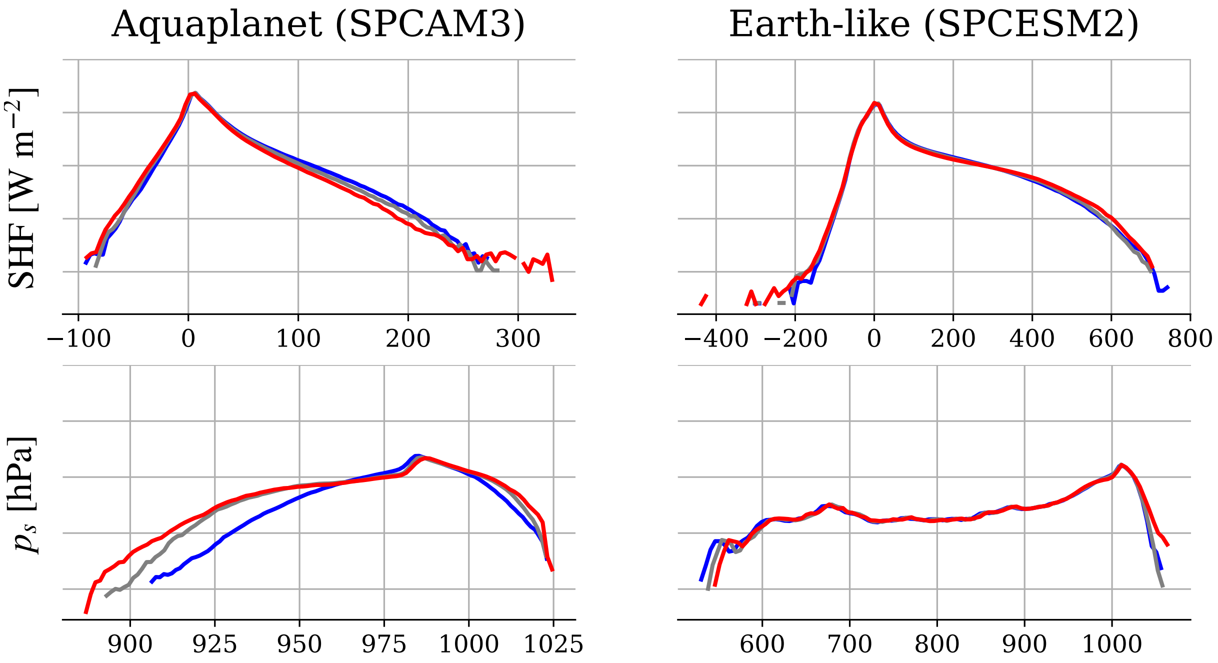


Figure S3: **Univariate PDFs of the sensible heat flux and surface pressure in the cold (blue), reference (gray), and warm (red) simulations of SPCAM3 and SPCESM2.** For a given variable, we use the same vertical logarithmic scale across models.

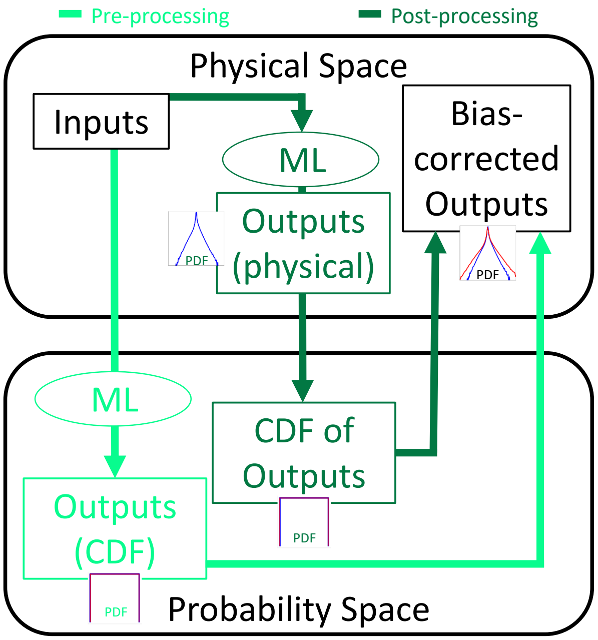


Figure S4: **Two types of bias-correction methods used to transform outputs in SM B2.** (1, dark green, "post-processing" method) Quantile mapping is typically done *after* training the model to bias-correct the outputs, and (2, light green, "pre-processing" method) we additionally test directly making predictions in probability space by converting the outputs to their CDF values *before* training. Note that this usually changes the loss function.

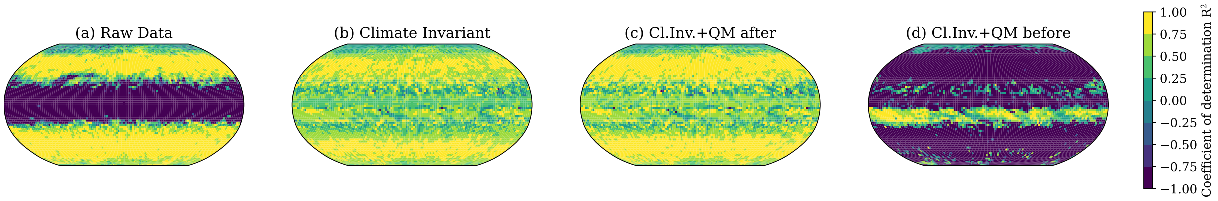


Figure S5: **Transforming outputs via quantile mapping *after* training slightly improves the climate invariant model's ability to generalize from a cold to a warm climate.** Coefficient of determination $R^2$ for 500-hPa subgrid heating of raw-data (a), climate-invariant (b), climate-invariant with outputs transformed *after* training (c), climate-invariant with outputs transformed *before* training (d) NNs trained using the cold (-4K) training set of SPCAM3 and calculated over the warm (+4K) training set of SPCAM3.

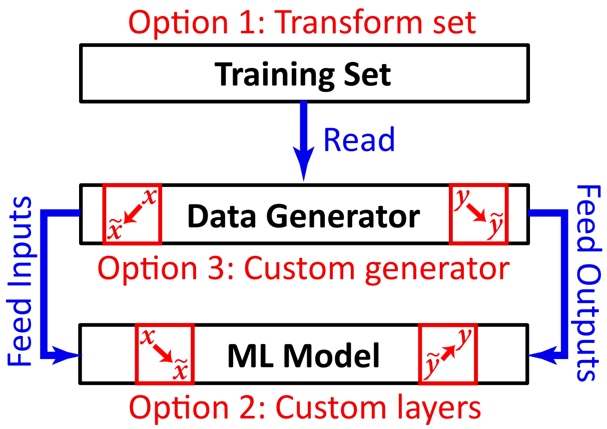


Figure S6: **Implementation of the climate-invariant ML framework.** The physical transformations can be implemented by (Option 1) transforming the training set, (Option 2) adding custom layers to the ML model, or (Option 3) customizing the data generator so that it automatically transforms the model inputs/outputs.

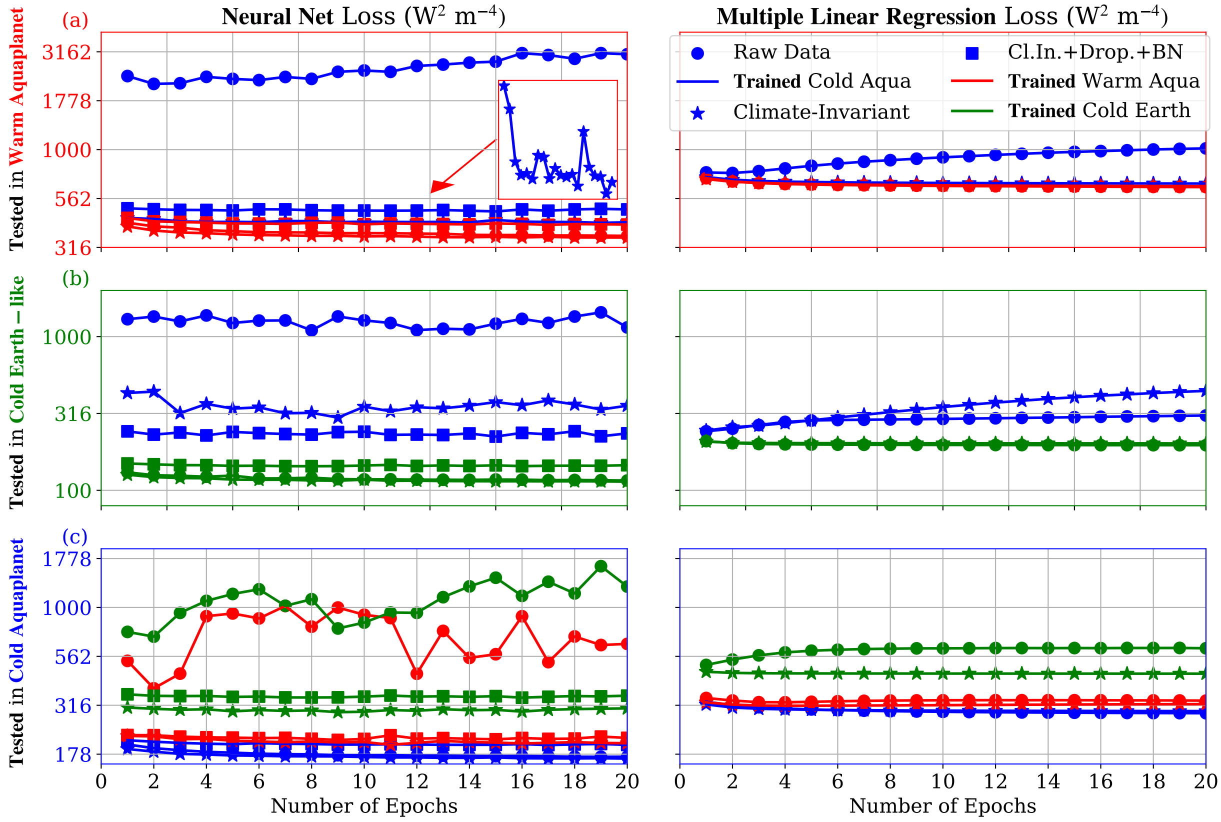


Figure S7: **Unlike raw-data models, climate-invariant models continuously learn about subgrid thermodynamics in the warm aquaplanet as they are trained in the cold aquaplanet.** More generally, they can learn information about configurations that differ from the one they were trained in. Learning curves of neural nets (left) and multiple linear regressions (right) **tested** in the (-4K) cold aquaplanet simulation (a, top row), the (+4K) warm aquaplanet simulation (b, middle row), and the (-4K) cold Earth-like simulation (c, bottom row). The lines' colors indicate the **training** dataset, while their symbols refer to whether the ML model is raw-data (circle), climate-invariant (star), or climate-invariant with dropout layers before each activation function and batch normalization (square). (b) We additionally zoom in on the climate-invariant neural network's learning curve in the (+4K) simulation.

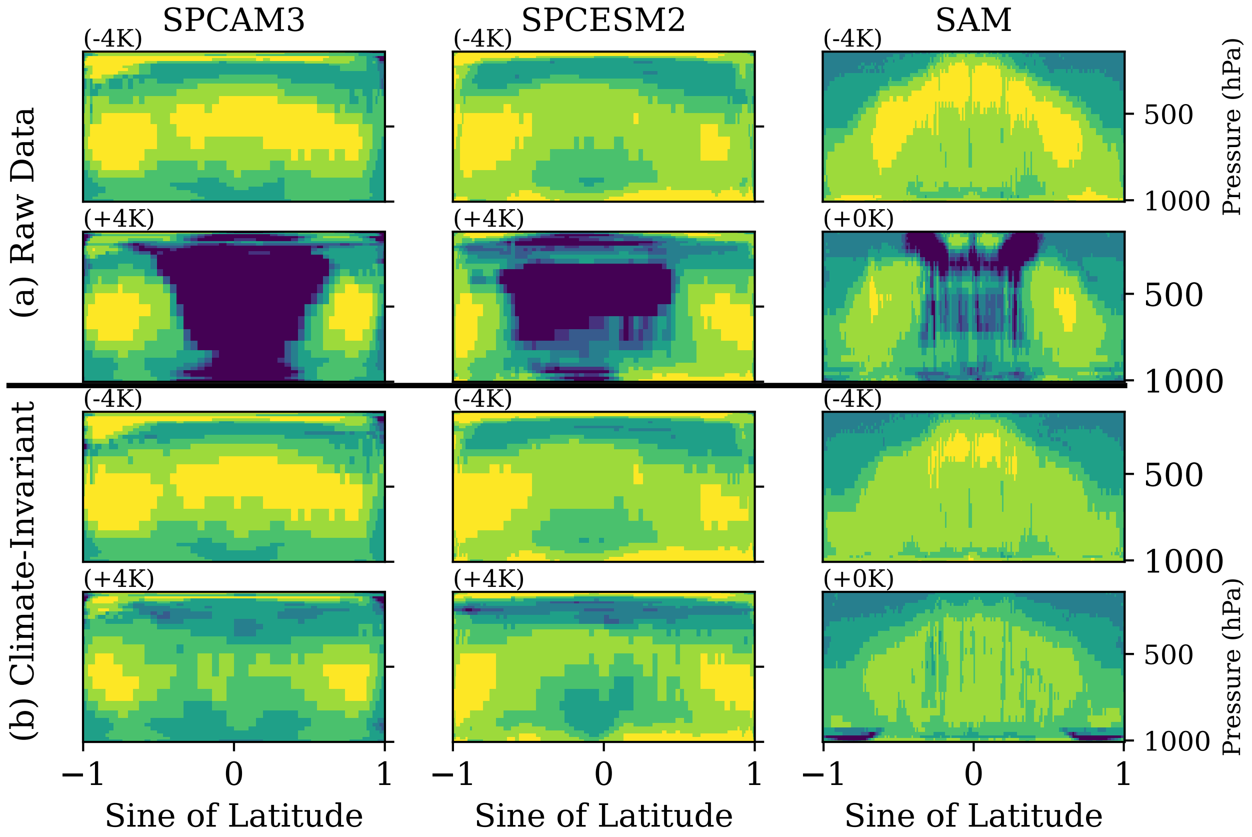


Figure S8: **Latitude-Pressure cross-section of subgrid heating's coefficient of determination $R^2$.** We train (a) raw-data and (b) climate-invariant NNs using the cold (-4K) training set of each model (SPCAM3, SPCESM2, and SAM) and test them in the cold (-4K) and warm (+4K) climates. See Fig 5 or Fig S9 for the colorbar.

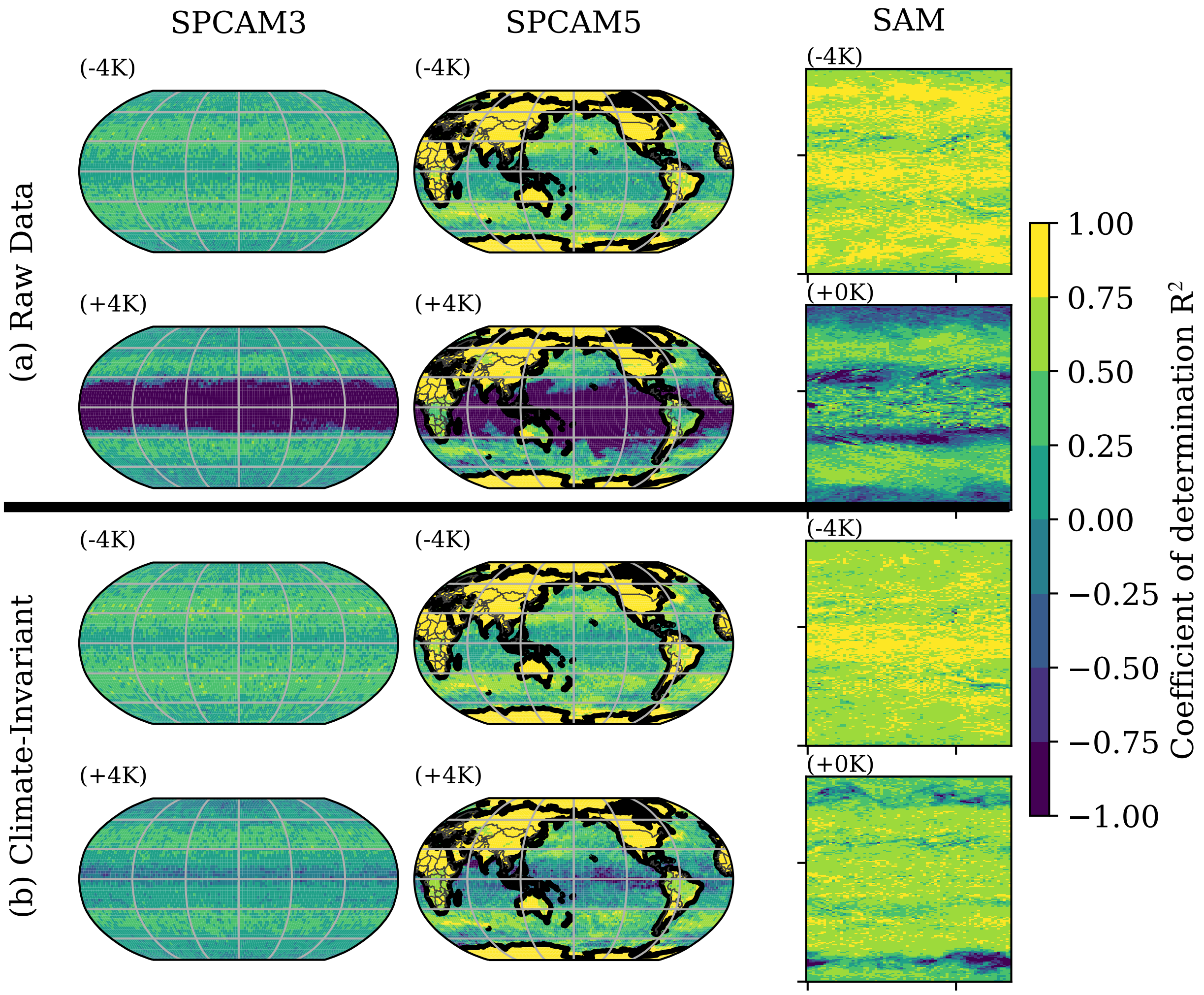


Figure S9: **Climate-invariant NNs mitigate generalization issues in the "Warm Tropics" for near-surface subgrid heating.** Same as Fig 6 for near-surface subgrid heating.

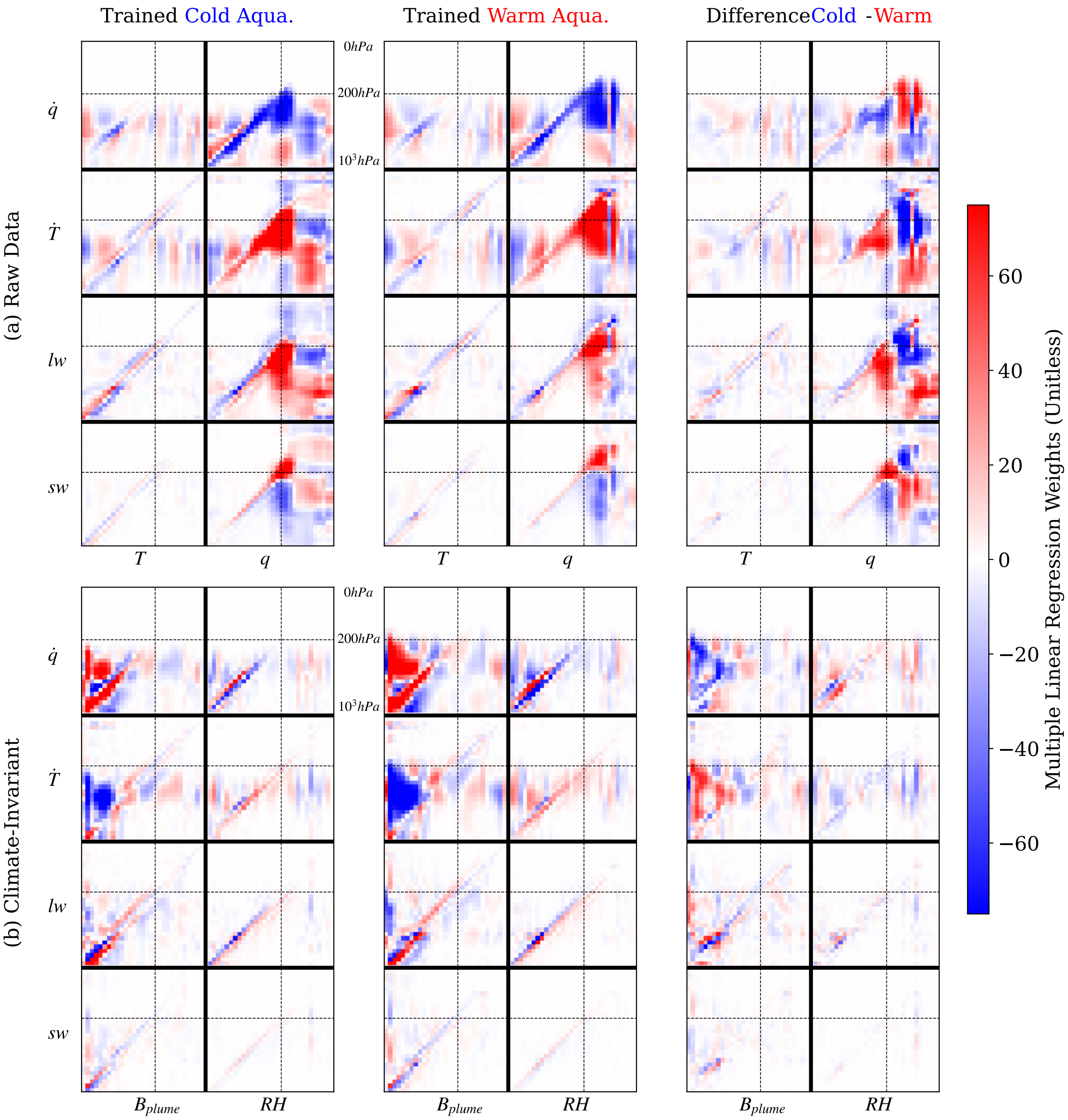


Figure S10: **Weights of the (a) raw-data and (b) climate-invariant multi-linear regressions trained in the cold (-4K) aquaplanet simulation (left), the warm (+4) warm aquaplanet simulation (middle), and their difference (right).** The x-axes indicate the vertical levels of the inputs, from the surface (left, $10^3$hPa) to the top of the atmosphere (right, 0hPa), while the y-axes indicate the vertical levels of the outputs, from the surface (bottom, $10^3$hPa) to the top of the atmosphere (top, 0hPa). We additionally indicate the 200hPa vertical level with dotted black lines.

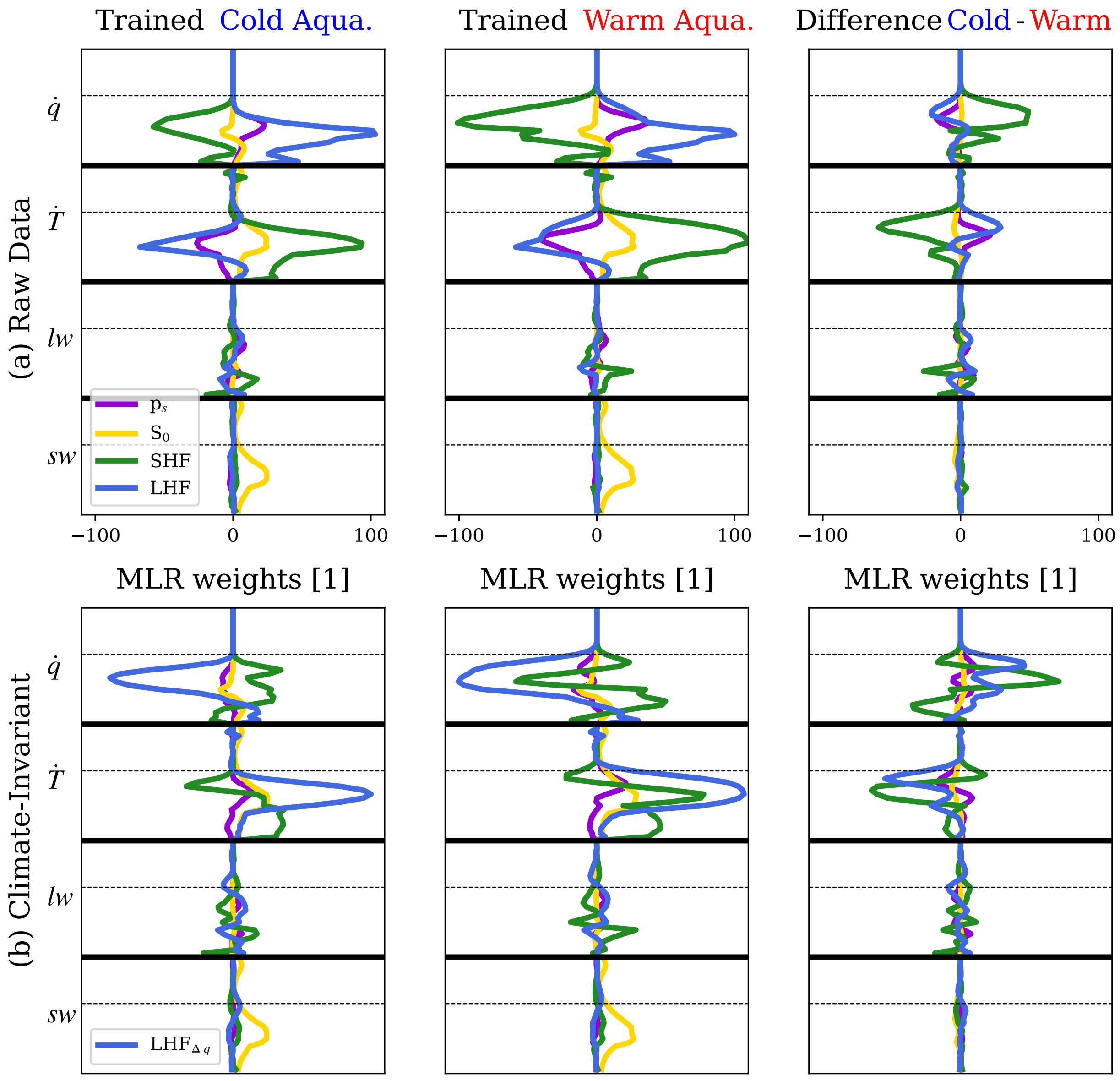


Figure S11: **Same as Fig S10, but for the four scalar inputs used in addition to the temperature and specific humidity i nputs.** The scalar inputs are surface pressure ($p_s$, purple line), solar insolation ($S_0$, yellow line), surface sensible heat flux (SHF, green line), and surface latent heat flux (LHF, blue line). For the climate-invariant mapping (b), LHF is transformed to $\mathrm{LHF}_{\Delta q}$ as described in Sec 3, which in conjunction with the temperature and humidity transformations, changes the multi-linear regression weights for all input variables.

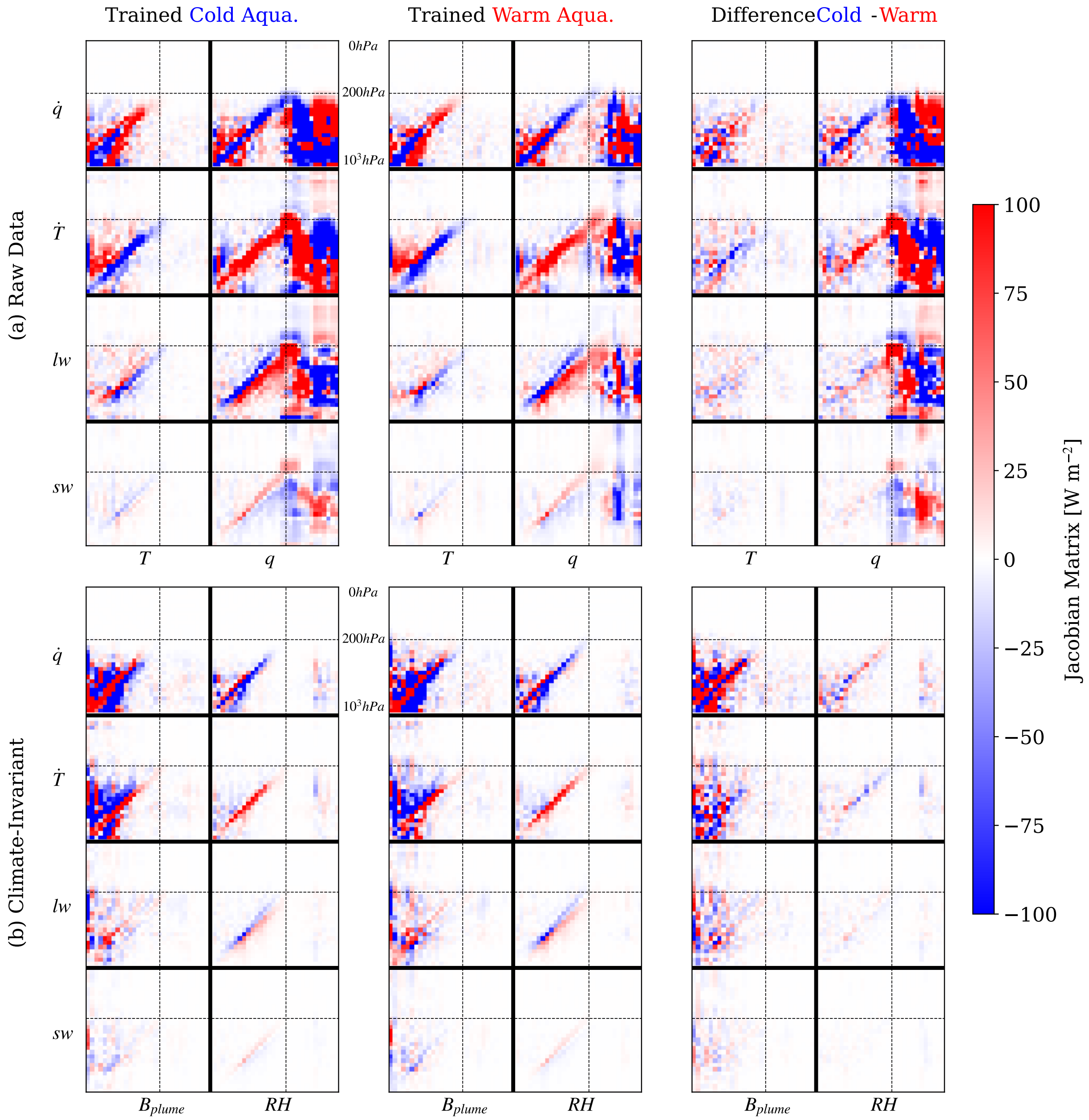


Figure S12: **Jacobian matrices of the (a) raw-data and (b) climate-invariant neural networks trained in the cold (-4K) aquaplanet simulation (left), the warm (+4) warm aquaplanet simulation (middle), and their difference (right).** The x-axes indicate the vertical levels of the inputs, from the surface (left, $10^3$hPa) to the top of the atmosphere (right, 0hPa), while the y-axes indicate the vertical levels of the outputs, from the surface (bottom, $10^3$hPa) to the top of the atmosphere (top, 0hPa). We additionally indicate the 200hPa vertical level with dotted black lines.

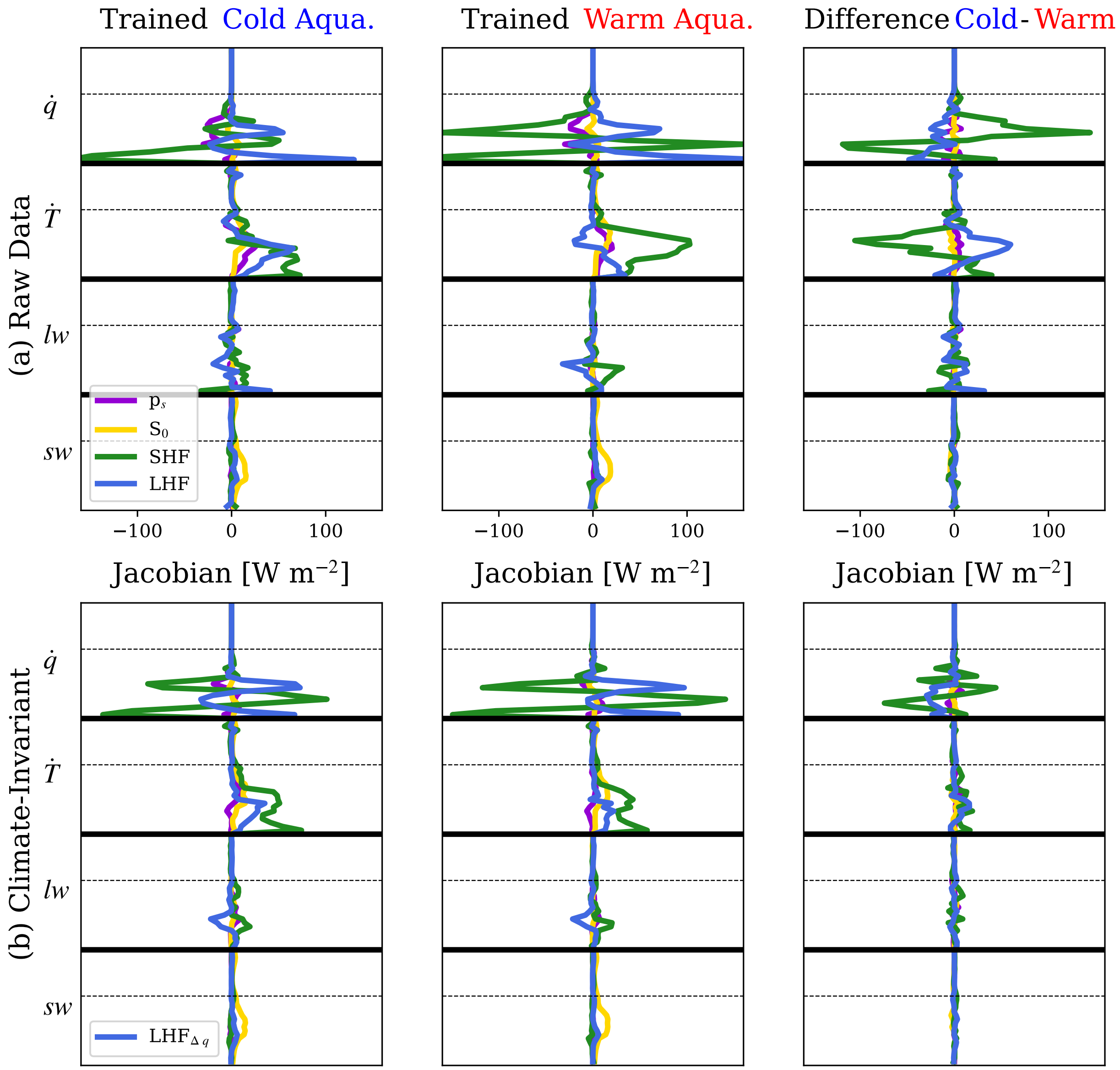


Figure S13: **Same as Fig S12, but for the four scalar inputs used in addition to the temperature and specific humidity i nputs.** The scalar inputs are surface pressure ($p_s$, purple line), solar insolation ($S_0$, yellow line), surface sensible heat flux (SHF, green line), and surface latent heat flux (LHF, blue line). For the climate-invariant mapping (b), LHF is transformed to $\mathrm{LHF}_{\Delta q}$ as described in Sec 3, which in conjunction with the temperature and humidity transformations, changes the Jacobian matrices for all input variables.

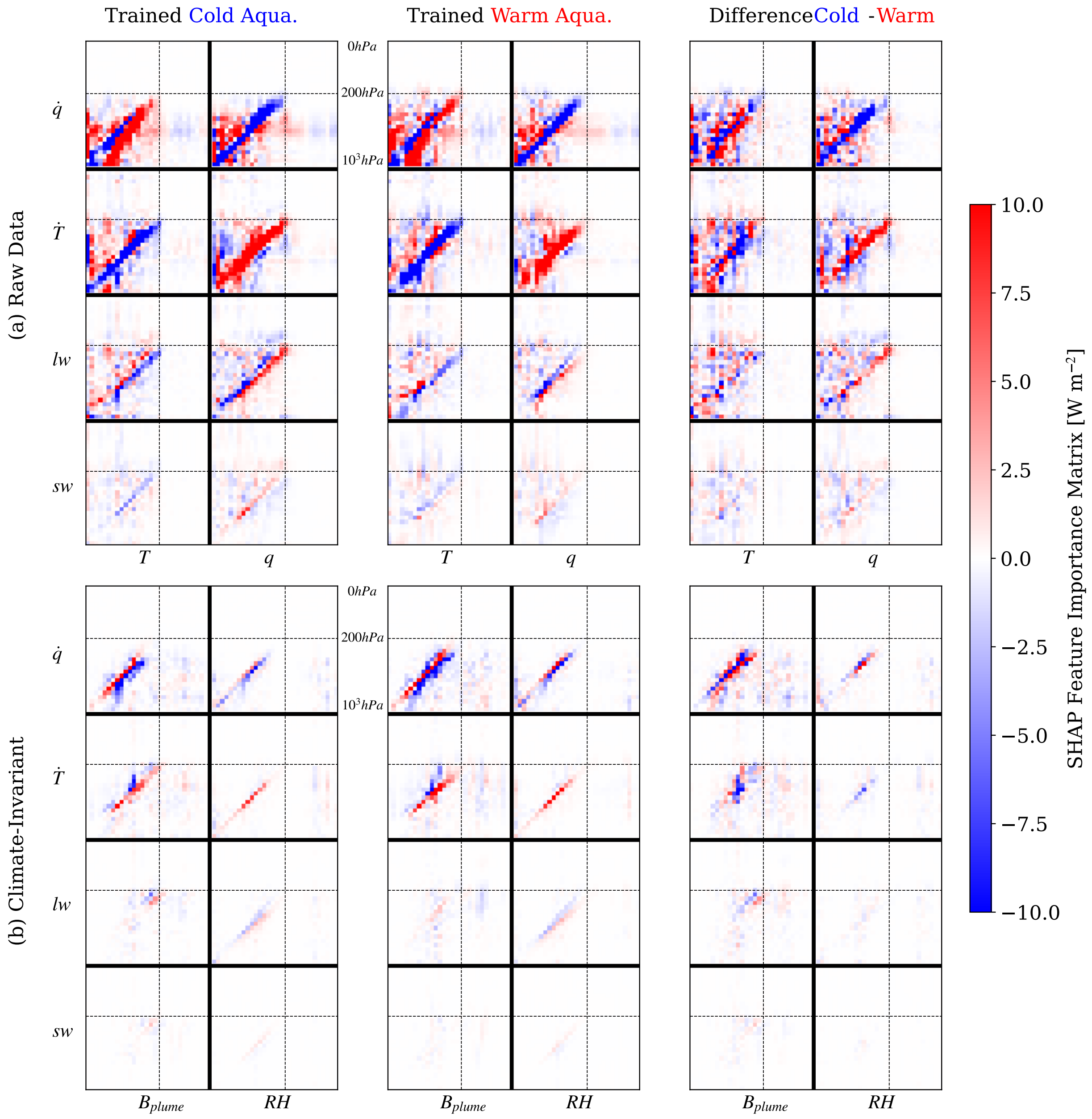


Figure S14: **SHAP feature importance matrix for the (a) raw-data and (b) climate-invariant neural nets trained in the cold (-4K) aquaplanet simulation (left), the warm (+4) warm aquaplanet simulation (middle), and their difference (right).** To calculate these matrices, we sample inputs from the (+4K) warm aquaplanet simulation for all ML models to facilitate inter-model comparison. The x-axes indicate the vertical levels of the inputs, from the surface (left, $10^3$hPa) to the top of the atmosphere (right, 0hPa), while the y-axes indicate the vertical levels of the outputs, from the surface (bottom, $10^3$hPa) to the top of the atmosphere (top, 0hPa). We additionally indicate the 200hPa vertical level with dotted black lines.

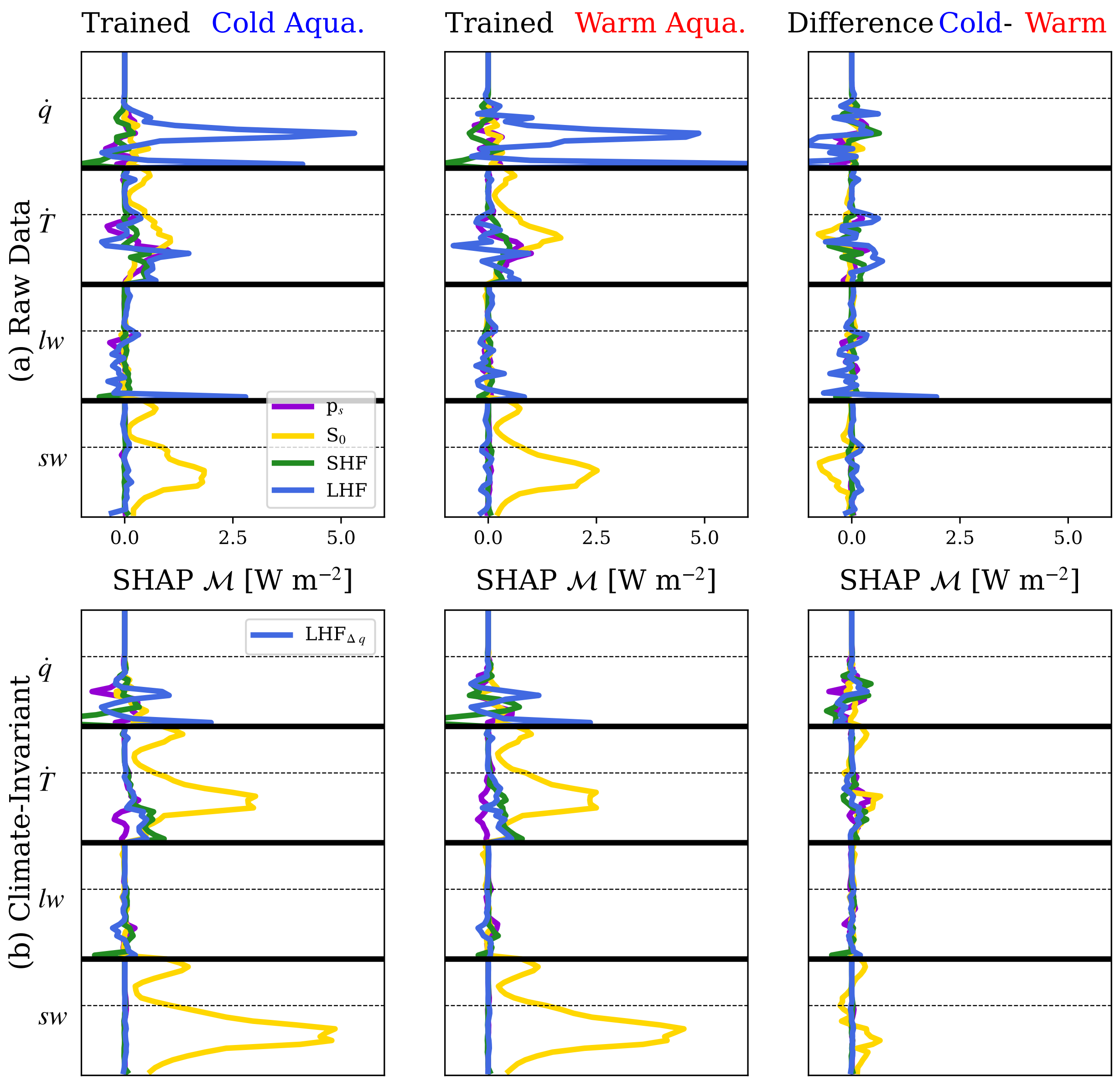


Figure S15: **Same as Fig S14, but for the four scalar inputs used in addition to the temperature and specific humidity inputs.** The scalar inputs are surface pressure ($p_s$, purple line), solar insolation ($S_0$, yellow line), surface sensible heat flux (SHF, green line), and surface latent heat flux (LHF, blue line). For the climate-invariant mapping (b), LHF is transformed to $\mathrm{LHF}_{\Delta q}$ as described in the "Theory" section, which in conjunction with the temperature and humidity transformations, changes the SHAP feature importance matrix for all input variables.

## REFERENCES


1. M. Reichstein, G. Camps-Valls, B. Stevens, M. Jung, J. Denzler, N. Carvalhais, Prabhat, Deep learning and process understanding for data-driven Earth system science. *Nature* **566**, 195–204 (2019).
2. T. Beucler, I. Ebert-Uphoff, S. Rasp, M. Pritchard, P. Gentine, "Machine learning for clouds and climate" in *Clouds and their Climatic Impacts: Radiation, Circulation, and Precipitation* (John Wiley & Sons, 2023), pp. 325–345.
3. C. Irrgang, N. Boers, M. Sonnewald, E. A. Barnes, C. Kadow, J. Staneva, J. Saynisch-Wagner, Towards neural Earth system modelling by integrating artificial intelligence in Earth system science. *Nat. Mach. Intell.* **3**, 667–674 (2021).
4. C. O. de Burgh-Day, T. Leeuwenburg, Machine learning for numerical weather and climate modelling: A review. *Geosci. Model Dev.* **16**, 6433–6477 (2023).
5. M. J. Molina, T. A. O'Brien, G. Anderson, M. Ashfaq, K. E. Bennett, W. D. Collins, K. Dagon, J. M. Restrepo, P. A. Ullrich, A review of recent and emerging machine learning applications for climate variability and weather phenomena. *Artif. Intell. Earth Syst.* **2**, 220086 (2023).
6. P. Ukkonen, R. Pincus, R. J. Hogan, K. P. Nielsen, E. Kaas, Accelerating radiation computations for dynamical models with targeted machine learning and code optimization. *J. Adv. Model. Earth Syst.* **12**, e2020MS002226 (2020).
7. A. Belochitski, P. Binev, R. De Vore, M. Fox-Rabinovitz, V. Krasnopolsky, P. Lamby, Tree approximation of the long wave radiation parameterization in the NCAR CAM global climate model. *J. Comput. Appl. Math.* **236**, 447–460 (2011).
8. J. J. Gristey, F. Graham, I. B. Glenn, K. Sebastian Schmidt, H. Chen, On the relationship between shallow cumulus cloud field properties and surface solar irradiance. *Geophys. Res. Lett.* **47**, e2020GL090152 (2020).
9. R. Lagerquist, D. Turner, I. Ebert-Uphoff, J. Stewart, V. Hagerty, Using deep learning to emulate and accelerate a radiative transfer model. *J. Atmos. Oceanic Tech.* **38**, 1673–1696 (2021).
10. M. Chantry, S. Hatfield, P. Dueben, I. Polichtchouk, T. Palmer, Machine learning emulation of gravity wave drag in numerical weather forecasting. *J. Adv. Model. Earth Syst.* **13**, e2021MS002477 (2021).
11. Z. I. Espinosa, A. Sheshadri, G. R. Cain, E. P. Gerber, K. J. Dalla Santa, Machine learning gravity wave parameterization generalizes to capture the QBO and response to increased $CO_2$. *Geophys. Res. Lett.* **49**, e2022GL098174 (2022).

12. D. Matsuoka, S. Watanabe, K. Sato, S. Kawazoe, W. Yu, S. Easterbrook, Application of deep learning to estimate atmospheric gravity wave parameters in reanalysis data sets. *Geophys. Res. Lett.* **47**, e2020GL089436 (2020).
13. J. Yuval, Paul A. O'Gorman, Neural-network parameterization of subgrid momentum transport in the atmosphere. *J. Adv. Model. Earth Syst.* **15**, e2023MS003606 (2023).
14. H. Morrison, M. van Lier-Walqui, M. R. Kumjian, O. P. Prat, A Bayesian approach for statistical-physical bulk parameterization of rain microphysics. Part I: Scheme description. *J. Atmos. Sci.* **77**, 1019–1041 (2019).
15. A. Seifert, S. Rasp, Potential and limitations of machine learning for modeling warm-rain cloud microphysical processes. *J. Adv. Model. Earth Syst.* **12**, e2020MS002301 (2020).
16. A. Gettelman, D. J. Gagne, C.-C. Chen, M. W. Christensen, Z. J. Lebo, H. Morrison, G. Gantos, Machine learning the warm rain process. *J. Adv. Model. Earth Syst.* **13**, e2020MS002268 (2021).
17. B. François, S. Thao, M. Vrac, Adjusting spatial dependence of climate model outputs with cycle-consistent adversarial networks. *Climate Dynam.* **57**, 3323–3353 (2021).
18. B. Pan, G. J. Anderson, A. Goncalves, D. D. Lucas, C. J. W. Bonfils, J. Lee, Y. Tian, H.-Y. Ma, Learning to correct climate projection biases. *J. Adv. Model. Earth Syst.* **13**, e2021MS002509 (2021).
19. V. Zantedeschi, F. Falasca, A. Douglas, R. Strange, M. J. Kusner, D. Watson-Parris, Cumulo: A dataset for learning cloud classes. arXiv:1911.04227 [quant-ph] (2019).
20. S. Rasp, H. Schulz, S. Bony, B. Stevens, Combining crowdsourcing and deep learning to explore the mesoscale organization of shallow convection. *Bull. Am. Meteorol. Soc.* **101**, E1980-E1995 (2020).
21. D. Watson-Parris, S. A. Sutherland, M. W. Christensen, R. Eastman, P. Stier, A large-scale analysis of pockets of open cells and their radiative impact. *Geophys. Res. Lett.* **48**, e2020GL092213 (2021).
22. L. Denby, Discovering the importance of mesoscale cloud organization through unsupervised classification. *Geophys. Res. Lett.* **47**, e2019GL085190 (2020).
23. Y. Han, G. J. Zhang, X. Huang, Y. Wang, A moist physics parameterization based on deep learning. *J. Adv. Model. Earth Syst.* **12**, e2020MS002076 (2020).
24. N. D. Brenowitz, C. S. Bretherton, Prognostic validation of a neural network unified physics parameterization. *Geophys. Res. Lett.* **45**, 6289–6298 (2018).
25. V. M. Krasnopolsky, M. S. Fox-Rabinovitz, A. A. Belochitski, Using ensemble of neural networks to learn stochastic convection parameterizations for climate and numerical weather prediction models

from data simulated by a cloud resolving model. *Advances in Artificial Neural Systems* **2013**, 485913 (2013).

26. L. Breiman, Random forests. *Mach. Learn.* **45**, 5–32 (2001).
27. P. A. O'Gorman, J. G. Dwyer, Using machine learning to parameterize moist convection: Potential for modeling of climate, climate change, and extreme events. *J. Adv. Model. Earth Syst.* **10**, 2548–2563 (2018).
28. A. Hernanz, Juan Andrés García-Valero, M. Domínguez, E. Rodríguez-Camino, A critical view on the suitability of machine learning techniques to downscale climate change projections: Illustration for temperature with a toy experiment. *Atmos. Sci. Lett.* **23**, e1087 (2022).
29. S. Rasp, M. S. Pritchard, P. Gentine, Deep learning to represent subgrid processes in climate models. *Proc. Natl. Acad. Sci.* **115**, 9684–9689 (2018).
30. T. Beucler, M. Pritchard, P. Gentine, S. Rasp, "Towards physically-consistent, data-driven models of convection" in *IGARSS 2020 - 2020 IEEE International Geoscience and Remote Sensing Symposium* (IEEE, 2020), pp. 3987–3990.
31. S. K. Clark, N. D. Brenowitz, B. Henn, A. Kwa, J. M. Gibbon, W. Andre Perkins, O. Watt-Meyer, C. S. Bretherton, L. M. Harris, Correcting a 200 km resolution climate model in multiple climates by machine learning from 25 km resolution simulations. *J. Adv. Model. Earth Syst.* **14**, e2022MS003219 (2022).
32. A. Doury, S. Somot, S. Gadat, A. Ribes, L. Corre, Regional climate model emulator based on deep learning: Concept and first evaluation of a novel hybrid downscaling approach. *Climate Dynam.* **60**, 1751–1779 (2023).
33. A. P. Guillaumin, L. Zanna, Stochastic-deep learning parameterization of ocean momentum forcing. *J. Adv. Model. Earth Syst.* **13**, e2021MS002534 (2021).
34. M. J. Molina, D. J. Gagne, A. F. Prein, A benchmark to test generalization capabilities of deep learning methods to classify severe convective storms in a changing *climate Earth Space Sci.* **8**, e2020EA001490 (2021).
35. Y. LeCun, Y. Bengio, "Convolutional networks for images, speech, and time series" *The Handbook of Brain Theory and Neural Networks* (MIT Press, 1995), p. 276.
36. S. Ioffe, C. Szegedy, "Batch normalization: Accelerating deep network training by reducing internal covariate shift" in *International Conference on Machine Learning* (PMLR, 2015), pp. 448–456.

37. N. Srivastava, G. Hinton, A. Krizhevsky, I. Sutskever, R. Salakhutdinov, Dropout: A simple way to prevent neural networks from overfitting. *J. Mach. Learn. Res.* **15**, 1929–1958 (2014).
38. X. Ying, An overview of overfitting and its solutions. *Journal of physics: Conference Series* **1168**, 022022 (2019).
39. G. Wilson, D. J. Cook, A survey of unsupervised deep domain adaptation. *ACM Trans. Intell. Syst. Technol.* **11**, 1–46 (2020).
40. V. M. Patel, R. Gopalan, R. Li, R. Chellappa, Visual domain adaptation: A survey of recent advances. *IEEE Signal Process. Mag.* **32**, 53–69 (2015).
41. D. Tuia, C. Persello, L. Bruzzone, Domain adaptation for the classification of remote sensing data: An overview of recent advances. *IEEE Trans. Geosci. Remote Sens.* **4**, 41–57 (2016).
42. S. J. Pan, Q. Yang, A survey on transfer learning. *IEEE Trans. Knowl. Data Eng.* **22**, 1345–1359 (2010).
43. W. M. Kouw, M. Loog, A review of domain adaptation without target labels. *IEEE Trans. Pattern Anal. Mach. Intell.* **43**, 766–785 (2021).
44. J. Ling, A. Kurzawski, J. Templeton, Reynolds averaged turbulence modelling using deep neural networks with embedded invariance. *J. Fluid Mech.* **807**, 155–166 (2016).
45. D. Randall, M. Khairoutdinov, A. Arakawa, W. Grabowski, Breaking the cloud parameterization deadlock. *Bull. Am. Meteorol. Soc.* **84**, 1547–1564 (2003).
46. S. Bony, B. Stevens, D. M. W. Frierson, C. Jakob, M. Kageyama, R. Pincus, T. G. Shepherd, S. C. Sherwood, A. Pier Siebesma, A. H. Sobel, M. Watanabe, M. J. Webb, Clouds, circulation and climate sensitivity. *Nat. Geosci.* **8**, 261–268 (2015).
47. T. Schneider, J. Teixeira, C. S. Bretherton, F. Brient, K. G. Pressel, C. Schär, A. Pier Siebesma, Climate goals and computing the future of *clouds Nat. Clim. Change* **7**, 3–5 (2017).
48. P. Gentine, V. Eyring, T. Beucler, "Deep learning for the parametrization of subgrid processes in climate models" in *Deep Learning for the Earth Sciences: A Comprehensive Approach to Remote Sensing, Climate Science, and Geosciences* (John Wiley & Sons, 2021), pp. 307–314.
49. N. D. Brenowitz, T. Beucler, M. Pritchard, C. S. Bretherton, Interpreting and stabilizing machine-learning parametrizations of convection. *J. Atmos. Sci.* **77**, 4357–4375 (2020).
50. J. Ott, M. Pritchard, N. Best, E. Linstead, M. Curcic, P. Baldi, A Fortran-Keras deep learning bridge for scientific computing. *Sci. Program.* **2020**8888811 (2020).

51. Forster, P., T. Storelvmo, K. Armour, W. Collins, J.-L. Dufresne, D. Frame, D. J. Lunt, T. Mauritsen, M. D. Palmer, M. Watanabe, M. Wild, H. Zhang, "The Earth's energy budget, climate feedbacks, and climate sensitivity" in *Climate Change 2021: The Physical Science Basis. Contribution of Working Group I to the Sixth Assessment Report of the Intergovernmental Panel on Climate Change*, V. Masson-Delmotte, P. Zhai, A. Pirani, S. L. Connors, C. Péan, S. Berger, N. Caud, Y. Chen, L. Goldfarb, M. I. Gomis, M. Huang, K. Leitzell, E. Lonnoy, J. B. R. Matthews, T. K. Maycock, T. Waterfield, O. Yelekçi, R. Yu, B. Zhou, Eds. (Cambridge Univ. Press, 2021), pp. 923–1054.
52. G. Mooers, M. Pritchard, T. Beucler, J. Ott, G. Yacalis, P. Baldi, P. Gentine, Assessing the potential of deep learning for emulating cloud superparameterization in climate models with real-geography boundary conditions. *J. Adv. Model. Earth Syst.* **13**, e2020MS002385 (2021).
53. P. Gentine, M. Pritchard, S. Rasp, G. Reinaudi, G. Yacalis, Could machine learning break the convection parameterization deadlock?. *Geophys. Res. Lett.* **45**, 5742–5751 (2018).
54. J. Yuval, P. A. O'Gorman, Stable machine-learning parameterization of subgrid processes for climate modeling at a range of resolutions. *Nat. Commun.* **11**, 3295 (2020).
55. J. Yuval, P. A. O'Gorman, C. N. Hill, Use of neural networks for stable, accurate and physically consistent parameterization of subgrid atmospheric processes with good performance at reduced precision. *Geophys. Res. Lett.* **48**, e2020GL091363 (2021).
56. M. Khairoutdinov, D. Randall, C. De Mott, Simulations of the atmospheric general circulation using a cloud-resolving model as a superparameterization of physical processes. *J. Atmos. Sci.* **62**, 2136–2154 (2005).
57. M. S. Pritchard, C. S. Bretherton, Causal evidence that rotational moisture advection is critical to the superparameterized Madden-Julian oscillation. *J. Atmos. Sci.* **71**, 800–815 (2014).
58. J. A. Andersen, Z. Kuang, Moist static energy budget of MJO-like disturbances in the atmosphere of a zonally symmetric aquaplanet. *J. Climate* **25**, 2782–2804 (2012).
59. M. F. Khairoutdinov, D. A. Randall, A cloud resolving model as a cloud parameterization in the NCAR Community Climate System Model: Preliminary results. *Geophys. Res. Lett.* **28**, 3617–3620 (2001).
60. T. Beucler, M. Pritchard, S. Rasp, J. Ott, P. Baldi, P. Gentine, Enforcing analytic constraints in neural networks emulating physical systems. *Phys. Rev. Lett.* **126**, 098302 (2021).

61. M. Wang, V. E. Larson, S. Ghan, M. Ovchinnikov, D. P. Schanen, H. Xiao, X. Liu, P. Rasch, Z. Guo, A multiscale modeling framework model (superparameterized CAM5) with a higher-order turbulence closure: Model description and low-cloud simulations. *J. Adv. Model. Earth Syst.* **7**, 484–509 (2015).
62. J. W. Hurrell, J. J. Hack, D. Shea, J. M. Caron, J. Rosinski, A new sea surface temperature and sea ice boundary dataset for the Community Atmosphere Model. *J. Climate* **21**, 5145–5153 (2008).
63. S. N. Tulich, A strategy for representing the effects of convective momentum transport in multiscale models: Evaluation using a new superparameterized version of the Weather Research and Forecast model (SP-WRF). *J. Adv. Model. Earth Syst.* **7**, 938–962 (2015).
64. N. D. Brenowitz, C. S. Bretherton, Spatially extended tests of a neural network parametrization trained by coarse-graining. *J. Adv. Model. Earth Syst.* **11**, 2728–2744 (2019).
65. T. Bolton, L. Zanna, Applications of deep learning to ocean data inference and subgrid parameterization. *J. Adv. Model. Earth Syst.* **11**, 376–399 (2019).
66. S. T. Garner, D. M. W. Frierson, I. M. Held, O. Pauluis, G. K. Vallis, Resolving convection in a global hypohydrostatic model. *J. Atmos. Sci.* **64**, 2061–2075 (2007).
67. W. R. Boos, A. Fedorov, L. Muir, Convective self-aggregation and tropical cyclogenesis under the hypohydrostatic rescaling. *J. Atmos. Sci.* **73**, 525–544 (2016).
68. P. A. O'Gorman, Z. Li, W. R. Boos, J. Yuval, Response of extreme precipitation to uniform surface warming in quasi-global aquaplanet simulations at high resolution. *Philos. Trans. A. Math. Phys. Eng. Sci.* **379**, 20190543 (2021).
69. R. B. Neale, B. J. Hoskins, A standard test for AGCMs including their physical parametrizations: I: The proposal. *Atmos. Sci. Lett.* **1**, 101–107 (2000).
70. A. Géron, *Hands-On Machine Learning With Scikit-Learn, Keras, and TensorFlow* (O'Reilly Media Inc., 2022).
71. A. Karpatne, I. Ebert-Uphoff, S. Ravela, H. A. Babaie, V. Kumar, Machine learning for the geosciences: Challenges and opportunities. *IEEE Trans. Knowl. Data Eng.* **31**, 1544–1554 (2019).
72. J. R. Holton, D. O. Staley, An introduction to dynamic meteorology. *Am. J. Phys.* **41**, 752–754 (1973).
73. A. Siebesma Pier, Sandrine Bony, Christian Jakob, Bjorn Stevens, Eds., *Clouds and Climate*: *Climate Science's Greatest Challenge* (Cambridge Univ. Press, 2020).
74. S. Manabe, R. T. Wetherald, Thermal equilibrium of the atmosphere with a given distribution of relative humidity. *J. Atmos. Sci.* **24**, 241–259 (1967).

75. R. G. Brown, C. Zhang, Variability of midtropospheric moisture and its effect on cloud-top height distribution during TOGA COARE. *J. Atmos. Sci.* **54**, 2760–2774 (1997).
76. C. S. Bretherton, M. E. Peters, L. E. Back, Relationships between water vapor path and precipitation over the tropical oceans. *J. Climate* **17**, 1517–1528 (2004).
77. C. E. Holloway, J. David Neelin, Temporal relations of column water vapor and tropical precipitation. *J. Atmos. Sci.* **67**, 1091–1105 (2010).
78. J. T. Seeley, N. Jeevanjee, D. M. Romps, FAT or FiTT: Are anvil clouds or the tropopause temperature invariant?. *Geophys. Res. Lett.* **46**, 1842–1850 (2019).
79. D. L. Hartmann, K. Larson, An important constraint on tropical cloud-climate feedback. *Geophys. Res. Lett.* **29**, 10.1029/2002GL015835 (2002).
80. Z. Kuang, D. L. Hartmann, Testing the fixed anvil temperature hypothesis in a cloud-resolving model. *J. Climate* **20**, 2051–2057 (2007).
81. F. Ahmed, J. David Neelin, Reverse engineering the tropical precipitation-buoyancy relationship. *J. Atmos. Sci.* **75**, 1587–1608 (2018).
82. F. Ahmed, Á. F. Adames, J. David Neelin, Deep convective adjustment of temperature and moisture. *J. Atmos. Sci.* **77**, 2163–2186 (2020).
83. K. A. Schiro, F. Ahmed, S. E. Giangrande, J David Neelin, GoAmazon2014/5 campaign points to deep-inflow approach to deep convection across scales. *Proc. Natl. Acad. Sci. U.S.A.* **115**, 4577–4582 (2018).
84. F. Ahmed, J. David Neelin, Protected convection as a metric of dry air influence on precipitation. *J. Climate* **34**, 3821–3838 (2021).
85. D. L. Hartmann, *Global Physical Climatology*, vol. 103 (Elsevier, 2015).
86. S. M. Lundberg, S.-I. Lee, A unified approach to interpreting model predictions. *Adv. Neural Inf. Process.* **30**, 4768–4777 (2017).
87. L. S. Shapley, “A value for n-person games” in *Contributions to the Theory of Games* (Princeton Univ. Press, 1953), pp. 307–317.
88. M. J. Herman, Z. Kuang, Linear response functions of two convective parameterization schemes. *J. Adv. Model. Earth Syst.* **5**, 510–541 (2013).
89. T. Beucler, T. Cronin, K. Emanuel, A linear response framework for radiative-convective instability. *J. Adv. Model. Earth Syst.* **10**, 1924–1951 (2018).

90. Z. Kuang, Linear stability of moist convecting atmospheres. Part I: From linear response functions to a simple model and applications to convectively coupled waves. *J. Atmos. Sci.* **75**, 2889–2907 (2018).
91. P.-J. Kindermans, S. Hooker, J. Adebayo, M. Alber, K. T. Schütt, S. Dähne, D. Erhan, B. Kim, "The (un) reliability of saliency methods" in *Explainable AI: Interpreting, Explaining and Visualizing Deep Learning* (Springer, 2019), pp. 267–280.
92. C. E. Holloway, J. David Neelin, The convective cold top and quasi equilibrium. *J. Atmos. Sci.* **64**, 1467–1487 (2007).
93. A. Zheng, A. Casari, *Feature Engineering for Machine Learning: Principles and Techniques for Data Scientists* (O'Reilly Media Inc., 2018).
94. T. G. Shepherd, Symmetries, conservation laws, and Hamiltonian structure in geophysical fluid dynamics. *Adv. Geophys.* **32**, 287–338 (1990).
95. R. Beran, Minimum Hellinger distance estimates for parametric models. *Ann. Stat.* **5**, 445–463 (1977).
96. D. M. Endres, J. E. Schindelin, A new metric for probability distributions. *IEEE Trans. Inf. Theory* **49**, 1858–1860 (2003).
97. S. Rasp, T. Beucler, G. Reinaudi, P. Gentine, tbeucler/CBRAIN-CAM: Climate-invariant branch, second release, Zenodo (2023); https://doi.org/10.5281/zenodo.8140413.
98. T. Beucler, M. Pritchard, L. Peng, J. Yuval, Climate-invariant machine learning, Zenodo (2023); https://doi.org/10.5281/zenodo.8140536.
99. S. Rasp, raspstephan/CBRAIN-CAM, Zenodo (2018); https://doi.org/10.5281/zenodo.1402384.
100. H. Parishani, M. S. Pritchard, C. S. Bretherton, C. R. Terai, M. C. Wyant, M. Khairoutdinov, B. Singh, Insensitivity of the cloud response to surface warming under radical changes to boundary layer turbulence and cloud microphysics: Results from the ultraparameterized CAM. *J. Adv. Model. Earth Syst.* **10**, 3139–3158 (2018).
101. J. Yuval, yaniyuval/Neural_nework_parameterization: Associated code and data for use of neural networks for stable, accurate and physically consistent parameterization of subgrid atmospheric processes with good performance at reduced precision, Zenodo (2021); https://doi.org/10.5281/zenodo.4118346.
102. M. F. Khairoutdinov, D. A. Randall, Cloud resolving modeling of the ARM summer 1997 IOP: Model formulation, results, uncertainties, and sensitivities. *J. Atmos. Sci.* **60**, 607–625 (2003).
103. K. A. Emanuel, *Atmospheric Convection* (Oxford Univ. Press, 1994).

104. Á. F. Adames, S. W. Powell, F. Ahmed, V. C. Mayta, J. David Neelin, Tropical precipitation evolution in a buoyancy-budget framework. *J. Atmos. Sci.* **78**, 509–528 (2021).

105. D. Maraun, Bias correcting climate change simulations – A critical review. *Curr. Clim. Change Rep.* **2**, 211–220 (2016).

106. N. Jeevanjee, D. M. Romps, Mean precipitation change from a deepening troposphere. *Proc. Natl. Acad. Sci.* **115**, 11465–11470 (2018).

107. M. S. Singh, Paul A. O'Gorman, Upward shift of the atmospheric general circulation under global warming: Theory and simulations. *J. Climate* **25**, 8259–8276 (2012).

108. D. Irving, A minimum standard for publishing computational results in the weather and climate sciences. *Bull. Am. Meteorol. Soc.* **97**, 1149–1158 (2016).

109. M. Abadi, A. Agarwal, P. Barham, E. Brevdo, Z. Chen, C. Citro, G. S. Corrado, A. Davis, J. Dean, M. Devin, S. Ghemawat, I. Goodfellow, A. Harp, G. Irving, M. Isard, Y. Jia, R. Jozefowicz, L. Kaiser, M. Kudlur, J. Levenberg, D. Mane, R. Monga, S. Moore, D. Murray, C. Olah, M. Schuster, J. Shlens, B. Steiner, I. Sutskever, K. Talwar, P. Tucker, V. Vanhoucke, V. Vasudevan, F. Viegas, O. Vinyals, P. Warden, M. Wattenberg, M. Wicke, Y. Yu, X. Zheng, TensorFlow: Large-scale machine learning on heterogeneous distributed systems. arXiv:1603.04467 (2016).

110. J. Kiefer, J. Wolfowitz, Stochastic estimation of the maximum of a regression function. *Ann. Math. Stat.* **23**, 462–466 (1952).

111. A. Paszke, S. Gross, F. Massa, A. Lerer, J. Bradbury, G. Chanan, T. Killeen, Z. Lin, N. Gimelshein, L. Antiga, A. Desmaison, A. Köpf, E. Yang, Z. De Vito, M. Raison, A. Tejani, S. Chilamkurthy, B. Steiner, L. Fang, J. Bai, S. Chintala, Pytorch: An imperative style, high-performance deep learning library. arXiv:1912.01703 (2019).

112. L. N. Smith, "Cyclical learning rates for training neural networks" in *2017 IEEE Winter Conference on Applications of Computer Vision (WACV)* (IEEE, 2017), pp. 464–472.

113. F. Chollet, Keras: The python deep learning library, Astrophysics Source Code Library, ascl-1806 (2018); https://keras.io/.
